# The establishment and application of quality gain-loss function when the loss of primary and cubic term is not ignored and the compensation quantity is constant

**DOI:** 10.1371/journal.pone.0295949

**Published:** 2023-12-18

**Authors:** Bo Wang, Xiaojuan Li, Jianyou Shi, Qikai Li, Xiangtian Nie

**Affiliations:** 1 School of Water Conservancy, North China University of Water Resources and Electric Power, Zhengzhou, China; 2 Collaborative Innovation Center of Water Resources Efficient Utilization and Protection Engineering, Zhengzhou, China; 3 Henan Key Laboratory of Water Environment Simulation and Treatment, Zhengzhou, China; 4 State Grid Xinyuan Central China Development & Construction Branch, Zhengzhou, China; University of Duhok, IRAQ

## Abstract

The traditional quality gain-loss function(QGLF) considers the case that the primary term loss cannot be ignored, does not consider the cubic term loss, but in practice the cubic term loss should not be ignored. In this paper, based on the existing QGLF model, the Taylor expansion is reserved to the third order, the determination of the quality loss coefficient is discussed and analyzed under the condition that the compensation quantity is constant, and the asymmetric cubic QGLF model is established, and uses an example of concrete mixture out of the machine slump during the dam concrete construction to analyze and discuss the relationship between the proposed model and the traditional quadratic QGLF, which verifies the rationality of the proposed model.

## 1 Introduction

In recent years, with the continuous intensification of market competition and the increasing demand of customers, the requirements for product quality are increasingly high, all walks of life pay more attention to product quality in a new height. Therefore, how to design and manufacture high quality and low cost products by quality engineering technology has attracted more and more attention from academia and other circles. In the early days, people believed that as long as the product quality was within the specification limit, it was a good product, and products beyond the specification limit were unqualified. In the early 1970s, Japanese quality management expert Dr. Taguchi [1], put forward the quality loss function, which connected the concepts of two different categories: quality and economy, and put forward the "Three designs", namely System design, Parameter design and Tolerance design. Since then, many scholars have done a lot of research on the basis of Taguchi quality loss theory [2, 3]. Spring et al. [4] proposed to solve the unbounded problem of the traditional quality loss function with the inverse normal distribution function, and established the asymmetric quality loss function model to solve the asymmetry problem of the quality loss function. Pan et al. [5] improved the limitation of symmetry in the Taguchi quality loss function, proposed a kind of non-symmetrical quality loss function, and based on this, constructed a comprehensive cost model that considers reworking cost, revising cost and Taguchi loss cost. Zhao et al. [6, 7] further extended Taguchi quality loss function and established piecewise quality loss function model by using piecewise function theory. Using the principle of capital discount, the service quality loss model was constructed. Fan et al. [8] studied a product quality model that took relative quality migration as an element and included multiple sub-quality indexes. Considering the interaction between each sub-quality index, they proposed a multivariate quality loss function model. Lee et al. [9] established the multiple quality loss model and discussed the tolerance design method. Zhang et al. [10] used likelihood uncorrelated regression technology to estimate the joint parameters of the model to consider the correlation between multiple responses, and proposed a multivariate expected quality loss function based on the weighted form of product index. Zhang et al. [11, 12] analyzed the problems existing in the quality loss function of smaller the better and larger the better characteristic, discussed the method of determining the loss coefficient of the primary and quadratic terms when the primary term loss cannot be ignored, proposed a quadratic quality loss function of smaller the better and larger the better characteristic without ignoring the loss of the primary term. Li et al. [13, 14] promoted Taylor’s expansion to the third order, discussed the quality loss function and established the cubic quality loss function. Mao et al. [15] proposed a quadratic exponential quality loss function and a new inherent reliability analysis method considering the different growth rates of quality characteristics when both sides of the target value deviate from the target value. In view of the fact that the traditional quality loss function cannot describe the quality compensation effect existing in production practice. Wang et al. [16–21] proposed the concept of quality gain-loss function(QGLF) on the basis of assigning the constant term in the Taylor series expansion as the meaning of quality compensation, and studied the quality gain-loss transfer model and the tolerance optimization method of quality characteristics. When the loss of primary term cannot be ignored and the compensation amount is constant, QGLF model of smaller the better and larger the better characteristic is designed.

QGLF considers that the loss caused by the deviation of product quality characteristics from the target value is the same and unbounded, which is inconsistent with many actual situations. At the same time, in the literature on QGLF, almost only one quadratic term is used to express the quality loss, which ignores both the primary term loss and the higher-order terms above the second order, which is inconsistent with some actual situations. Therefore, this paper studies the cubic QGLF and its application without ignoring the loss of primary term and cubic term when the quality compensation is constant.

## 2 The nominal-the-type characteristic QGLF

Let *y* be the quality characteristic value of the product (For example, concrete mixture outlet temperature, outlet slump, air content of concrete, etc.), and *y*_*0*_ be the target value for product performance design, respectively, and the corresponding quality gain-loss of *y* be *G(y)*. Assuming that *G(y)* has a second derivative at *y = y*_*0*_, we can expand it according to Taylor’s expansion:

$$G(y)=G(y_{0})+\frac{G\prime (y_{0})}{1!}(y-y_{0})+\frac{G\prime \prime (y_{0})}{2!}{\left(y-y_{0}\right)}^{2}+o{\left(y-y_{0}\right)}^{2}$$ (1)

When *y = y*_*0*_, the quality characteristic value equals to target value, and QGLF *G(y)* achieves the minimum *G(y*_*0*_*)*, *i*.*e*. *G*′(*y*_0_) = 0. Since there is quality compensation, *G*(*y*_0_) ∈ *R*. Ignoring higher order terms above second order, we get:

$$G(y)=G(y_{0})+k_{2}{\left(y-y_{0}\right)}^{2}$$ (2)

**Fig 1:**
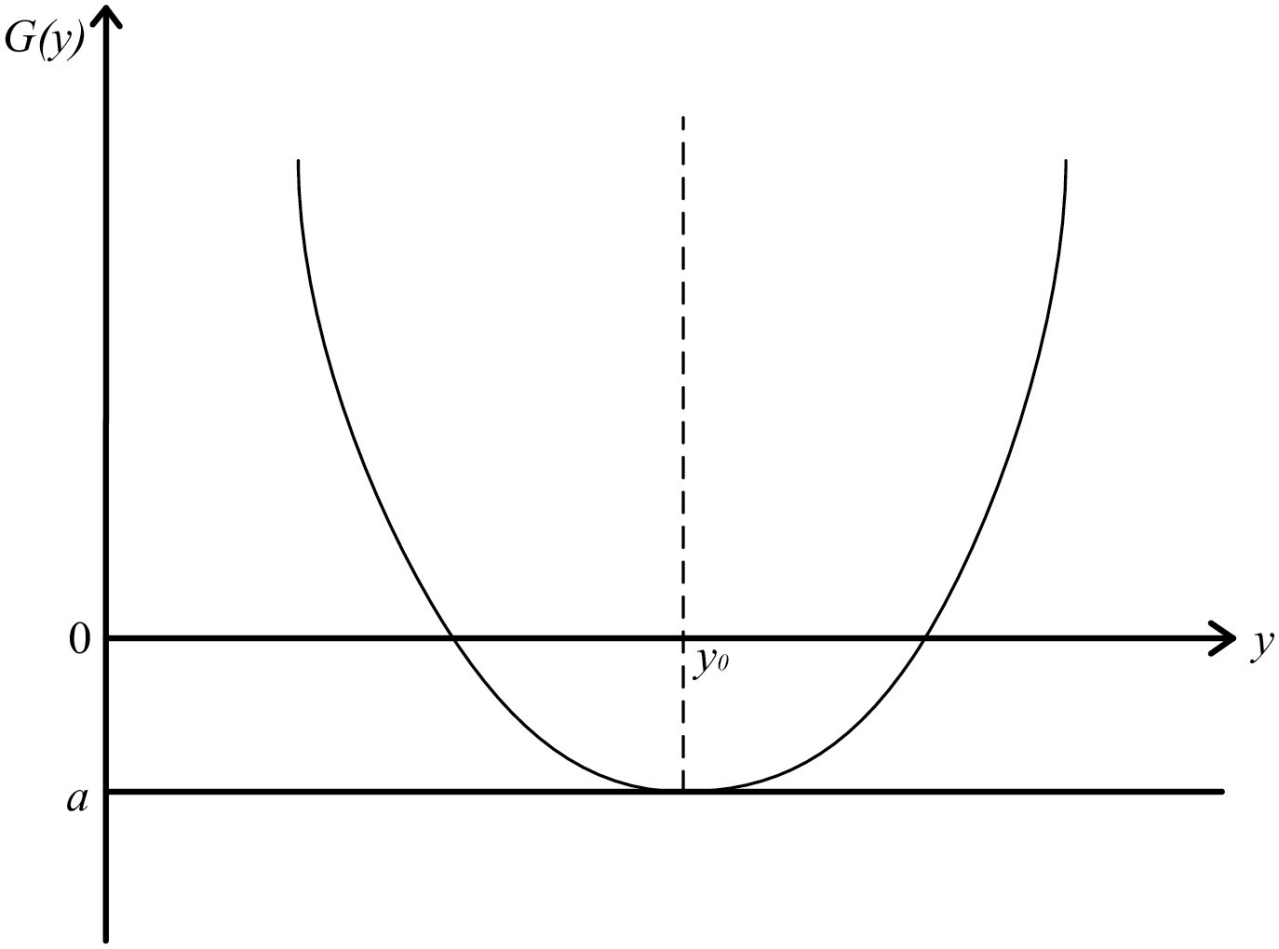
Image of QGLF (*G(y*_*0*_*)<0*).

Where, *k*_*2*_ represents the quality loss coefficient, *k*_2_ = *G*″(*y*_0_)/2! is a constant; *G(y*_*0*_*)* represents the quality compensation quantity, which is assumed to be represented by a constant a. Since the loss function has the minimum quality loss when *y = y*_*0*_, a can represent the maximum quality gain. The image of QGLF(*G(y*_*0*_*)<0*) is shown in Fig 1.

Eq (2) represents QGLF when the quality compensation is constant, and *G(y)* represents the corresponding quality gain-loss when the product characteristic value is y. When *G(y)*<0, the total quality gain, i.e. the quality loss caused by fluctuation, is less than the quality compensation; when *G(y)*>0, the total quality loss, i.e. the quality loss caused by fluctuation, is greater than the quality compensation; when *G(y)* = 0, i.e. the quality loss caused by the fluctuation is equal to the quality compensation, and the gain-loss is 0. Assume that the functional boundary of product quality characteristics is *Δ*, and the quality gain-loss for loss of function is *G*_*0*_, i.e.


$$G_{0}=a+k_{2}\Delta^{2}$$ (3)

Assume that the specification limit of product quality characteristics is *ζ* and the nonconformity quality gain-loss is *G*, i.e.


$$G=a+k_{2}\zeta^{2}$$ (4)

The quality loss coefficient *k*_*2*_ can be determined from Eqs (3) or (4), i.e.

$$k_{2}=\frac{G_{0}-a}{\Delta^{2}}=\frac{G-a}{\zeta^{2}}$$ (5)

(*G*_0_ − *a*)*ζ*^2^ = (*G* − *a*)Δ^2^ can be obtained from Eq (5). Therefore, it can be concluded that the quadratic QGLF must satisfy the condition (*G*_0_ − *a*)*ζ*^2^ = (*G* − *a*)Δ^2^, which limits the application of the function. In order to expand its application scope, QGLF should be further studied.

## 3 The cubic QGLF design

Assuming *G(y)* is differentiable in the third order at *y = y*_*0*_, the Taylor expansion is retained to the cubic term, and the loss of the primary term is not ignored, then the cubic QGLF can be defined as:

$$G(y)=a+k_{1}(y-y_{0})+k_{2}{\left(y-y_{0}\right)}^{2}+k_{3}{\left(y-y_{0}\right)}^{3}$$ (6)

Where, *k*_*1*_, *k*_*2*_ and *k*_*3*_ respectively represent the quality loss coefficient of the primary term, the quality loss coefficient of the quadratic term and the quality loss coefficient of the cubic term, all of which are constants independent of *y*. *a* is a constant and the quality compensation quantity.

Under normal circumstances, the quality loss caused by product quality characteristics located on both sides of the target value is asymmetric. In actual production, the requirements for quality characteristics on two sides of the target value are not necessarily the same. For example, when producing parts, if the part size is greater than the maximum size required by the functional limit of the part, the part size can be qualified through rework, and the quality loss caused at this time is the cost of rework; however, if the size of the produced part is less than the minimum size required by the functional limits of the part, the part can only be treated as scrap, and the quality loss caused by this case is the value of the part itself. Therefore, QGLF represented by the piecewise function when the quality compensation is constant is as follows:

$$G_{N}(y)=\left\{\begin{array}{ll} a+A_{01} & ,y<y_{0}-\Delta_{1}\\ a+k_{1}(y-y_{0})+k_{2}{\left(y-y_{0}\right)}^{2}+k_{3}{\left(y-y_{0}\right)}^{3} & ,y_{0}-\Delta_{1}\leq y<y_{0}\\ a+k_{4}(y-y_{0})+k_{5}{\left(y-y_{0}\right)}^{2}+k_{6}{\left(y-y_{0}\right)}^{3} & ,y_{0}\leq y\leq y_{0}+\Delta_{2}\\ a+A_{02} & ,y>y_{0}+\Delta_{2} \end{array}\right.$$ (7)

**Fig 2:**
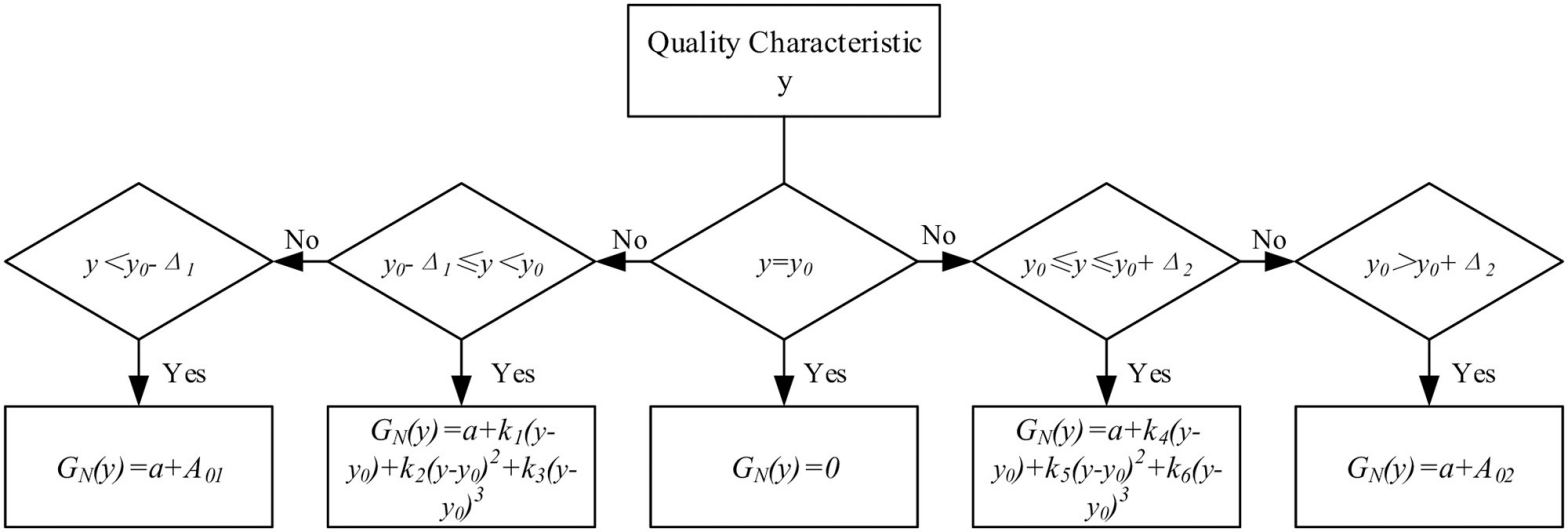
Flow chart of the cubic QGLF.

In this formula, *G*_*N*_*(y)* represents the quality gain-loss of the product, *y*_*0*_*-Δ*_*1*_ and *y*_*0*_*+Δ*_*2*_ are the functional limits of product quality characteristics, *A*_*01*_ and *A*_*02*_ are the quality loss values, *A*_*01*_*a+A*_*01*_ and *a+A*_*02*_ are the quality gain-loss values, *k*_*1*_, *k*_*2*_ and *k*_*3*_ are the quality loss coefficients of primary term, quadratic term and cubic term on the left of the target value, respectively. *k*_*4*_, *k*_*5*_ and *k*_*6*_ are the quality loss coefficients of primary term, quadratic term and cubic term on the right of the target value, respectively (Fig 2).

Different from the quadratic QGLF, the cubic QGLF has three quality loss coefficients on both sides of the target value. How to find the relationship between the coefficients and get the correct solution of each coefficient has become a problem that must be solved to determine the cubic QGLF.

Assuming that *y*_*0*_*-Δ*_*1*_ and *y*_*0*_*+Δ*_*2*_ are the lower and upper functional limits of product quality characteristics respectively, the corresponding gain-loss are *a+A*_*01*_ and *a+A*_*02*_, respectively. Beyond the functional limits, the product can be regarded as losing its function. *y*_*0*_*-ζ*_*1*_ and *y*_*0*_*+ζ*_*2*_ are the lower and upper specification limits of product quality characteristics, and the corresponding gain-loss are a+A1 and a+A2, respectively. Beyond the specification limits, the product can be regarded as unqualified. It can be obtained as follows:

$$a-k_{1}\Delta_{1}+k_{2}\Delta_{1}^{2}-k_{3}\Delta_{1}^{3}=a+A_{01}$$ (8)

$$a-k_{1}\zeta_{1}+k_{2}\zeta_{1}^{2}-k_{3}\zeta_{1}^{3}=a+A_{1}$$ (9)

$$a+k_{4}\zeta_{2}+k_{5}\zeta_{2}^{2}+k_{6}\zeta_{2}^{3}=a+A_{2}$$ (10)

$$a+k_{4}\Delta_{2}+k_{5}\Delta_{2}^{2}+k_{6}\Delta_{2}^{3}=a+A_{02}$$ (11)

In the following two cases, the relationship between the quality loss coefficients *k*_*1*_, *k*_*2*_, *k*_*3*_ and *k*_*4*_, *k*_*5*_, *k*_*6*_ is discussed in the ranges [*y*_0_ − Δ_1_, *y*_0_] and [*y*_0_, *y*_0_ + Δ_2_], respectively.

Let

$$G_{N1}(y)=a+k_{1}(y-y_{0})+k_{2}{\left(y-y_{0}\right)}^{2}+k_{3}{\left(y-y_{0}\right)}^{3}$$ (12)

$$L_{N1}(y)=k_{1}(y-y_{0})+k_{2}{\left(y-y_{0}\right)}^{2}+k_{3}{\left(y-y_{0}\right)}^{3}$$ (13)

In the formula, a is a constant, representing the quality compensation quantity; *L*_*N1*_*(y)* represents the quality loss; *y* ∈ [*y*_0_ − Δ_1_, *y*_0_].

So *G*_*N*1_(*y*) = *a* + *L*_*N*1_(*y*).

Let *G*_*N*1_(*y*) = *a*, which is *L*_*N*1_(*y*) = 0, and get

$$\left\{\begin{array}{l} y_{1}=y_{0}\\ y_{2}=\frac{-k_{2}+2k_{3}y_{0}+\sqrt{k_{2}^{2}-4k_{1}k_{3}}}{2k_{3}}\\ y_{3}=\frac{-k_{2}+2k_{3}y_{0}-\sqrt{k_{2}^{2}-4k_{1}k_{3}}}{2k_{3}} \end{array}\right.$$ (14)

By observing the upper solution, it can be seen that *k*_3_ ≠ 0 and $k_{2}^{2}-4k_{1}k_{3}\geq 0$, i.e. $k_{2}^{2}\geq 4k_{1}k_{3}$. If *k*_*3*_ = 0, the original equation becomes a quadratic quality loss function, i.e. the cubic term loss is 0, and the cubic term loss is not considered. If $k_{2}^{2}-4k_{1}k_{3}=0$, the cubic equation has two zero solutions; if $k_{2}^{2}-4k_{1}k_{3}>0$, the equation has three zero solutions.

Let

$$G_{N2}(y)=a+k_{4}(y-y_{0})+k_{5}{\left(y-y_{0}\right)}^{2}+k_{6}{\left(y-y_{0}\right)}^{3}$$ (15)

$$L_{N2}(y)=k_{4}(y-y_{0})+k_{5}{\left(y-y_{0}\right)}^{2}+k_{6}{\left(y-y_{0}\right)}^{3}$$ (16)

In the formula, a is a constant, representing the quality compensation quantity; *L*_*N2*_*(y)* represents the quality loss; *y* ∈ [*y*_0_, *y*_0_ + Δ_2_].

So *G*_*N*2_(*y*) = *a* + *L*_*N*2_(*y*).

Let *G*_*N*2_(*y*) = *a*, which is *L*_*N*2_(*y*) = 0, and get

$$\left\{\begin{array}{l} y_{6}=y_{0}\\ y_{7}=\frac{-k_{5}+2k_{6}y_{0}+\sqrt{k_{5}^{2}-4k_{4}k_{6}}}{2k_{6}}\\ y_{8}=\frac{-k_{5}+2k_{6}y_{0}-\sqrt{k_{5}^{2}-4k_{4}k_{6}}}{2k_{6}} \end{array}\right.$$ (17)

By observing the upper solution, it can be seen that *k*_6_ ≠ 0 and $k_{5}^{2}-4k_{4}k_{6}\geq 0$, i.e. $k_{5}^{2}\geq 4k_{4}k_{6}$. If *k*_*6*_ = 0, the original equation becomes a quadratic quality loss function, i.e. the cubic term loss is 0, and the cubic term loss is not considered. If $k_{5}^{2}-4k_{4}k_{6}=0$, the cubic equation has two zero solutions; if $k_{5}^{2}-4k_{4}k_{6}>0$, the equation has three zero solutions.

This paper first considers the discriminant is equal to 0, and then analyzes the discriminant is greater than or equal to 0, in order to extend the application of cubic QGLF to the general.

### 3.1 The discriminant is 0

#### 3.1.1 When y ∈ *[y*_*0*_ *− Δ*_*1*_, y_*0*_*]*

Let $k_{2}^{2}-4k_{1}k_{3}=0$, so $k_{2}=2\sqrt{k_{1}k_{3}}$ or $k_{2}=-2\sqrt{k_{1}k_{3}}$.

*3*.*1*.*1*.*1 When $k_{2}=2\sqrt{k_{1}k_{3}}$*.

According to Eqs (8) and (9) and $k_{2}=2\sqrt{k_{1}k_{3}}$, it can be solved

$$\begin{array}{l} k_{1}=-\frac{A_{01}\zeta_{1}^{3}+A_{1}\Delta_{1}^{3}-2\Delta_{1}\zeta_{1}\sqrt{A_{01}A_{1}\Delta_{1}\zeta_{1}}}{\Delta_{1}\zeta_{1}{\left(\Delta_{1}-\zeta_{1}\right)}^{2}}=-\frac{{\left(\sqrt{A_{01}\zeta_{1}^{3}}-\sqrt{A_{1}\Delta_{1}^{3}}\right)}^{2}}{\Delta_{1}\zeta_{1}{\left(\Delta_{1}-\zeta_{1}\right)}^{2}}\\ k_{2}=\frac{2\sqrt{{\left(\sqrt{A_{01}\zeta_{1}^{3}}-\sqrt{A_{1}\Delta_{1}^{3}}\right)}^{2}{\left(\sqrt{A_{01}\zeta_{1}}-\sqrt{A_{1}\Delta_{1}}\right)}^{2}}}{\Delta_{1}\zeta_{1}{\left(\Delta_{1}-\zeta_{1}\right)}^{2}}\\ k_{3}=-\frac{A_{01}\zeta_{1}+A_{1}\Delta_{1}-2\sqrt{A_{01}A_{1}\Delta_{1}\zeta_{1}}}{\Delta_{1}\zeta_{1}{\left(\Delta_{1}-\zeta_{1}\right)}^{2}}=-\frac{{\left(\sqrt{A_{01}\zeta_{1}}-\sqrt{A_{1}\Delta_{1}}\right)}^{2}}{\Delta_{1}\zeta_{1}{\left(\Delta_{1}-\zeta_{1}\right)}^{2}} \end{array}$$ (18)

or

$$\begin{array}{l} k_{1}=-\frac{A_{01}\zeta_{1}^{3}+A_{1}\Delta_{1}^{3}+2\Delta_{1}\zeta_{1}\sqrt{A_{01}A_{1}\Delta_{1}\zeta_{1}}}{\Delta_{1}\zeta_{1}{\left(\Delta_{1}-\zeta_{1}\right)}^{2}}=-\frac{{\left(\sqrt{A_{01}\zeta_{1}^{3}}+\sqrt{A_{1}\Delta_{1}^{3}}\right)}^{2}}{\Delta_{1}\zeta_{1}{\left(\Delta_{1}-\zeta_{1}\right)}^{2}}\\ k_{2}=\frac{2[A_{01}\zeta_{1}^{2}+A_{1}\Delta_{1}^{2}+(\Delta_{1}+\zeta_{1})\sqrt{A_{01}A_{1}\Delta_{1}\zeta_{1}}]}{\Delta_{1}\zeta_{1}{\left(\Delta_{1}-\zeta_{1}\right)}^{2}}\\ k_{3}=-\frac{A_{01}\zeta_{1}+A_{1}\Delta_{1}+2\sqrt{A_{01}A_{1}\Delta_{1}\zeta_{1}}}{\Delta_{1}\zeta_{1}{\left(\Delta_{1}-\zeta_{1}\right)}^{2}}=-\frac{{\left(\sqrt{A_{01}\zeta_{1}}+\sqrt{A_{1}\Delta_{1}}\right)}^{2}}{\Delta_{1}\zeta_{1}{\left(\Delta_{1}-\zeta_{1}\right)}^{2}} \end{array}$$ (19)

It can be seen from Eqs (18) and (19) that *k*_*1*_≤0, *k*_*2*_≥0, *k*_*3*_<0. Since *Δ*_*1*_≥0, *ζ*_*1*_≥0, according to Eq (8), $k_{2}=2\sqrt{k_{1}k_{3}}$ and the basic inequality, we can obtain

$$\frac{-k_{1}\Delta_{1}-k_{3}\Delta_{1}^{3}}{2}\geq \sqrt{\left(-k_{1}\Delta_{1})(-k_{3}\Delta_{1}^{3}\right)}=\Delta_{1}^{2}\sqrt{k_{1}k_{3}}$$

$$∴k_{2}\leq \frac{A_{01}}{2\Delta_{1}^{2}}$$ (20)

Similarly, according to Eq (9), we can obtain

$$k_{2}\leq \frac{A_{1}}{2\zeta_{1}^{2}}$$ (21)

The value range of *k*_*2*_ can be obtained by adding Eqs (20) and (21)

$$0\leq k_{2}\leq \frac{A_{01}\zeta_{1}^{2}+A_{1}\Delta_{1}^{2}}{2\Delta_{1}^{2}\zeta_{1}^{2}}$$ (22)

According to Eqs (19) and (22), we can obtain

$$k_{2}=\frac{2[A_{01}\zeta_{1}^{2}+A_{1}\Delta_{1}^{2}+(\Delta_{1}+\zeta_{1})\sqrt{A_{01}A_{1}\Delta_{1}\zeta_{1}}]}{\Delta_{1}\zeta_{1}{\left(\Delta_{1}-\zeta_{1}\right)}^{2}}\leq \frac{A_{01}\zeta_{1}^{2}+A_{1}\Delta_{1}^{2}}{2\Delta_{1}^{2}\zeta_{1}^{2}}$$

i.e.


$$\left[4\Delta_{1}\zeta_{1}-{\left(\Delta_{1}-\zeta_{1}\right)}^{2}\right](A_{01}\zeta_{1}^{2}+A_{1}\Delta_{1}^{2})+2\Delta_{1}\zeta_{1}(\Delta_{1}+\zeta_{1})\sqrt{A_{01}A_{1}\Delta_{1}\zeta_{1}}\leq 0$$ (23)

Assume Δ_1_ ≥ 1, *ζ*_1_ ≥1, 0 ≤ Δ_1_ − *ζ*_1_ ≤ 1, at this time 4Δ_1_*ζ*_1_ ≥ 4, (Δ_1_ − *ζ*_1_)^2^ ≤ 1, so 4Δ_1_*ζ*_1_ − (Δ_1_ − *ζ*_1_)^2^ ≥ 0, in this case

$$\left[4\Delta_{1}\zeta_{1}-{\left(\Delta_{1}-\zeta_{1}\right)}^{2}\right](A_{01}\zeta_{1}^{2}+A_{1}\Delta_{1}^{2})+2\Delta_{1}\zeta_{1}(\Delta_{1}+\zeta_{1})\sqrt{A_{01}A_{1}\Delta_{1}\zeta_{1}}\geq 0$$ (24)

This is contrary to Eq (23), so the solution of the coefficient corresponding to Eq (19) should be removed.

The solution of quality loss coefficient corresponding to Eq (18) is analyzed as follows. *L*_*N*1_(*y*) = 0 has two zero solutions, which are *y*_1_ = *y*_0_ and *y*_2,3_ = *y*_0_ − *k*_2_/2*k*_3_. Because *k*_1_ ≤ 0, *k*_2_ ≥ 0, *k*_3_ < 0, so *k*_2_/2*k*_3_ ≤ 0, that is *y*_2,3_ > *y*_1_.

*L*_*N*1_(*y*) calculates the first derivative of *y*, we can get

$$L_{N1}^{\prime }(y)=k_{1}+k_{2}(y-y_{0})+k_{3}{\left(y-y_{0}\right)}^{2}$$ (25)

**Fig 3:**
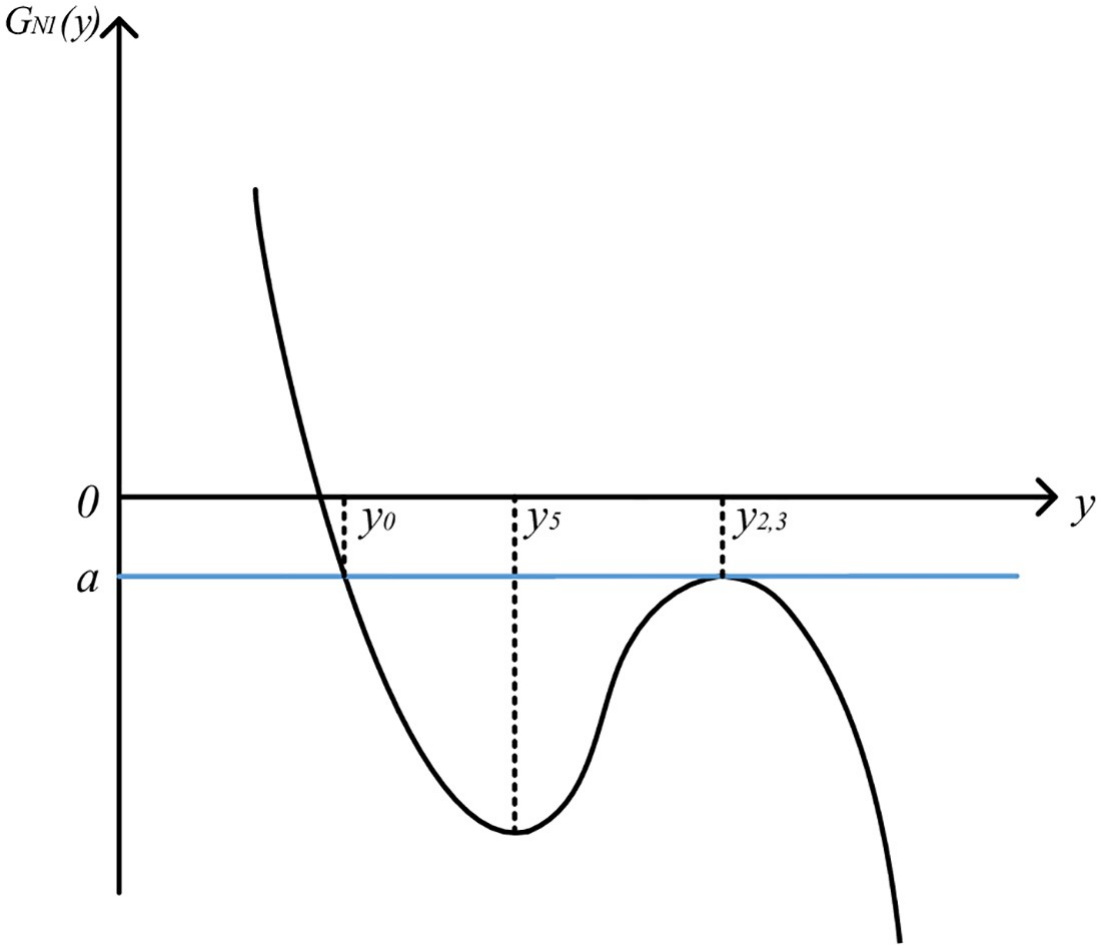
Image of QGLF *G*_*N*1_(*y*) ($k_{2}=2\sqrt{k_{1}k_{3}}$).

Let $L_{N1}^{\prime }(y)=0$, the solution is *y*_4_ = *y*_0_ − *k*_2_/2*k*_3_ = *y*_2,3_, *y*_5_ = *y*_0_ − *k*_2_/6*k*_3_. Because *k*_2_ ≥ 0 and *k*_3_ < 0, so *y*_2,3_ > *y*_5_ > *y*_0_. Therefore, the image of function *L*_*N*1_(*y*) is roughly in the form of "subtraction, increase and subtraction". At this time, the image of QGLF *G*_*N*1_(*y*) is shown in Fig 3.

From the QGLF image, it can be seen that when *y* ∈ [*y*_0_ − Δ_1_, *y*_0_], the function image is monotonous, and the quality gain-loss in this case is consistent with the actual product quality gain-loss. Therefore, when $k_{2}=2\sqrt{k_{1}k_{3}}$, QGLF *G*_*N*1_(*y*) on the interval [*y*_0_ − Δ_1_, *y*_*0*_] can be formulated as

$$\begin{array}{l} G_{N1}(y)=a-\frac{{\left(\sqrt{A_{01}\zeta_{1}^{3}}-\sqrt{A_{1}\Delta_{1}^{3}}\right)}^{2}}{\Delta_{1}\zeta_{1}{\left(\Delta_{1}-\zeta_{1}\right)}^{2}}(y-y_{0})\\ +\frac{2\sqrt{{\left(\sqrt{A_{01}\zeta_{1}^{3}}-\sqrt{A_{1}\Delta_{1}^{3}}\right)}^{2}{\left(\sqrt{A_{01}\zeta_{1}}-\sqrt{A_{1}\Delta_{1}}\right)}^{2}}}{\Delta_{1}\zeta_{1}{\left(\Delta_{1}-\zeta_{1}\right)}^{2}}{\left(y-y_{0}\right)}^{2}\\ -\frac{{\left(\sqrt{A_{01}\zeta_{1}}-\sqrt{A_{1}\Delta_{1}}\right)}^{2}}{\Delta_{1}\zeta_{1}{\left(\Delta_{1}-\zeta_{1}\right)}^{2}}{\left(y-y_{0}\right)}^{3} \end{array}$$ (26)

**Fig 4:**
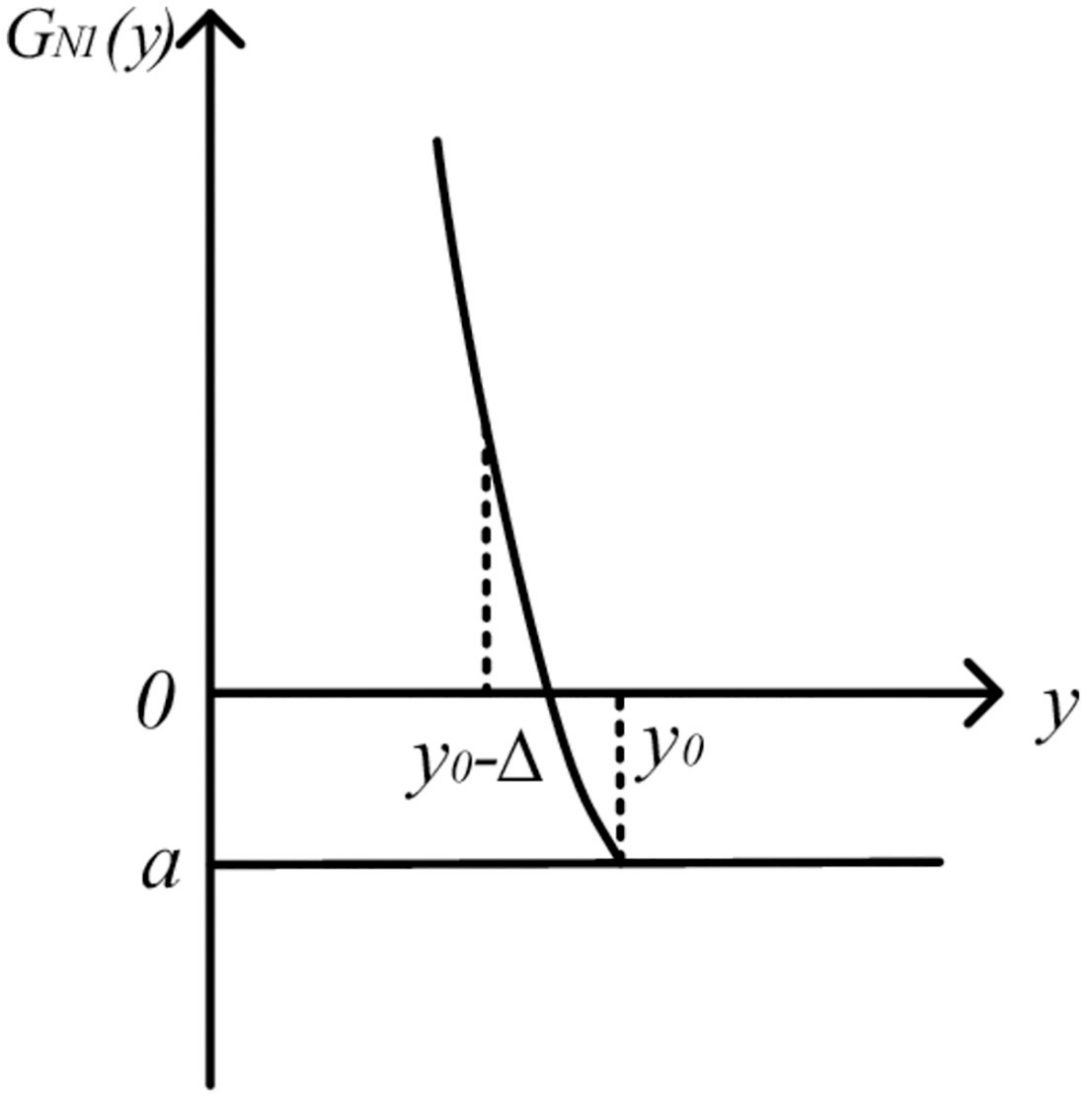
Image of QGLF *G*_*N*1_(*y*) (*y* ∈ [*y*_0_ − Δ_1_, *y*_0_) and $k_{2}=2\sqrt{k_{1}k_{3}}$).

The image is shown in Fig 4.

*3*.*1*.*1*.*2 When $k_{2}=-2\sqrt{k_{1}k_{3}}$*.

According to Eqs (8), (9) and $k_{2}=-2\sqrt{k_{1}k_{3}}$, it can be solved

$$\begin{array}{l} k_{1}=-\frac{{\left(\sqrt{A_{01}\zeta_{1}^{3}}-\sqrt{A_{1}\Delta_{1}^{3}}\right)}^{2}}{\Delta_{1}\zeta_{1}{\left(\Delta_{1}-\zeta_{1}\right)}^{2}}\\ k_{2}=-\frac{2\sqrt{{\left(\sqrt{A_{01}\zeta_{1}^{3}}-\sqrt{A_{1}\Delta_{1}^{3}}\right)}^{2}{\left(\sqrt{A_{01}\zeta_{1}}-\sqrt{A_{1}\Delta_{1}}\right)}^{2}}}{\Delta_{1}\zeta_{1}{\left(\Delta_{1}-\zeta_{1}\right)}^{2}}\\ k_{3}=-\frac{{\left(\sqrt{A_{01}\zeta_{1}}-\sqrt{A_{1}\Delta_{1}}\right)}^{2}}{\Delta_{1}\zeta_{1}{\left(\Delta_{1}-\zeta_{1}\right)}^{2}} \end{array}$$ (27)

or

$$\begin{array}{l} k_{1}=-\frac{{\left(\sqrt{A_{01}\zeta_{1}^{3}}+\sqrt{A_{1}\Delta_{1}^{3}}\right)}^{2}}{\Delta_{1}\zeta_{1}{\left(\Delta_{1}-\zeta_{1}\right)}^{2}}\\ k_{2}=-\frac{2[A_{01}\zeta_{1}^{2}+A_{1}\Delta_{1}^{2}+(\Delta_{1}+\zeta_{1})\sqrt{A_{01}A_{1}\Delta_{1}\zeta_{1}}]}{\Delta_{1}\zeta_{1}{\left(\Delta_{1}-\zeta_{1}\right)}^{2}}\\ k_{3}=-\frac{{\left(\sqrt{A_{01}\zeta_{1}}+\sqrt{A_{1}\Delta_{1}}\right)}^{2}}{\Delta_{1}\zeta_{1}{\left(\Delta_{1}-\zeta_{1}\right)}^{2}} \end{array}$$ (28)

It can be seen that *k*_1_ ≤ 0, *k*_2_ ≤ 0, *k*_3_ < 0, at this point *y*_2,3_ < *y*_5_ < *y*_0_. To determine the trend of function image, the size relationship between *y*_2,3_ and 0 should be analyzed.


$$\begin{array}{l} ∵\;\frac{k_{2}}{2k_{3}}=[-\frac{2\sqrt{{\left(\sqrt{A_{01}\zeta_{1}^{3}}-\sqrt{A_{1}\Delta_{1}^{3}}\right)}^{2}{\left(\sqrt{A_{01}\zeta_{1}}-\sqrt{A_{1}\Delta_{1}}\right)}^{2}}}{\Delta_{1}\zeta_{1}{\left(\Delta_{1}-\zeta_{1}\right)}^{2}}]\cdot [-\frac{\Delta_{1}\zeta_{1}{\left(\Delta_{1}-\zeta_{1}\right)}^{2}}{{\left(\sqrt{A_{01}\zeta_{1}}-\sqrt{A_{1}\Delta_{1}}\right)}^{2}}]\\ =\sqrt{\frac{{\left(\sqrt{A_{01}\zeta_{1}^{3}}-\sqrt{A_{1}\Delta_{1}^{3}}\right)}^{2}}{{\left(\sqrt{A_{01}\zeta_{1}}-\sqrt{A_{1}\Delta_{1}}\right)}^{2}}}=\sqrt{{\left(\frac{\zeta_{1}\sqrt{A_{01}\zeta_{1}}-\Delta_{1}\sqrt{A_{1}\Delta_{1}}}{\sqrt{A_{01}\zeta_{1}}-\sqrt{A_{1}\Delta_{1}}}\right)}^{2}}<\sqrt{{\left[\frac{\Delta_{1}(\sqrt{A_{01}\zeta_{1}}-\Delta_{1}\sqrt{A_{1}\Delta_{1}})}{\sqrt{A_{01}\zeta_{1}}-\sqrt{A_{1}\Delta_{1}}}\right]}^{2}}=\Delta_{1} \end{array}$$

$$∴y_{0}-\frac{k_{2}}{2k_{3}}>y_{0}-\Delta_{1}$$ (29)

$$∵\;y_{0}-\Delta_{1}>0$$

$$∴y_{0}-\frac{k_{2}}{6k_{3}}>y_{0}-\frac{k_{2}}{2k_{3}}>0$$

**Fig 5:**
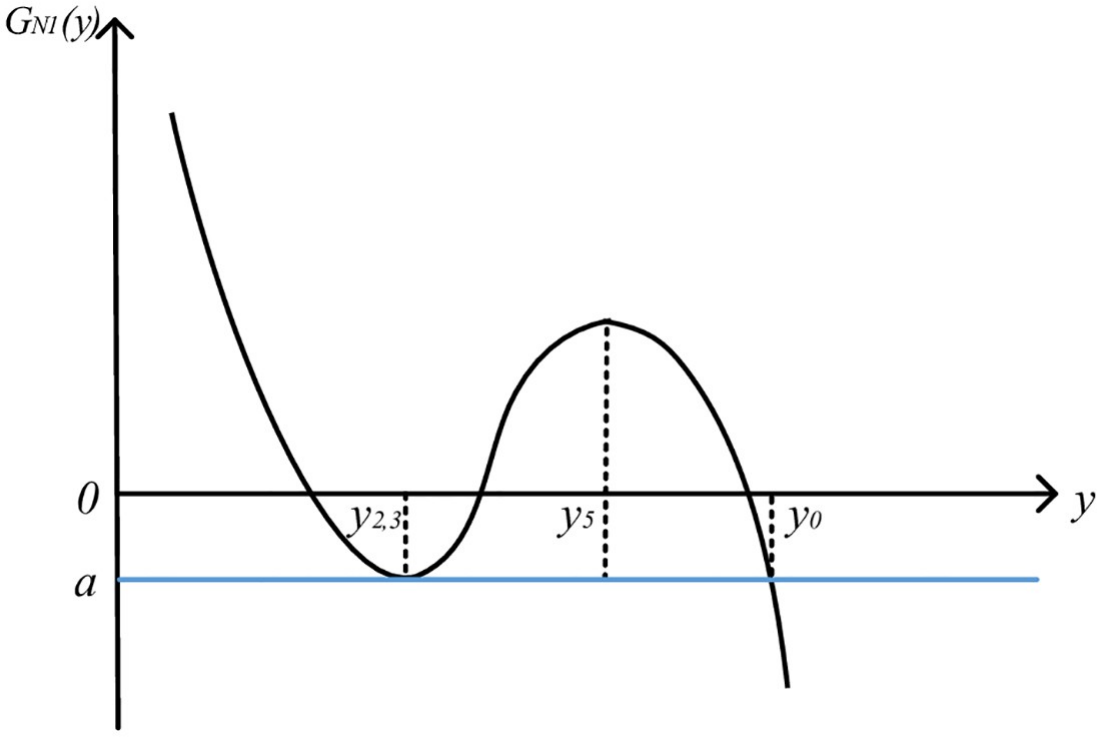
Image of QGLF *G*_*N*1_(*y*) ($k_{2}=-2\sqrt{k_{1}k_{3}}$).

Therefore, *y*_*2*,*3*_>0. In this case, the image of QGLF is shown in Fig 5.

From the QGLF image, it can be seen that when *y* ∈ [*y*_0_ − Δ_1_, *y*_0_], the function image is not monotonous, and the quality gain-loss in this case is not consistent with the actual product quality gain-loss. Therefore, when $k_{2}=-2\sqrt{k_{1}k_{3}}$, the obtained QGLF does not conform to the reality and should be discarded.

**3.1.2 When** *y* ∈ [*y*_0_, *y*_0_ +Δ_2_]

Let $k_{5}^{2}-4k_{4}k_{6}=0$, so $k_{5}=2\sqrt{k_{4}k_{6}}$ or $k_{5}=-2\sqrt{k_{4}k_{6}}$.

*3*.*1*.*2*.*1 When $k_{5}=2\sqrt{k_{4}k_{6}}$*.

According to Eqs (10) and 11 and $k_{5}=2\sqrt{k_{4}k_{6}}$, it can be solved

$$\begin{array}{l} k_{4}=\frac{A_{02}\zeta_{2}^{3}+A_{2}\Delta_{2}^{3}-2\Delta_{2}\zeta_{2}\sqrt{A_{02}A_{2}\Delta_{2}\zeta_{2}}}{\Delta_{2}\zeta_{2}{\left(\Delta_{2}-\zeta_{2}\right)}^{2}}=\frac{{\left(\sqrt{A_{02}\zeta_{2}^{3}}-\sqrt{A_{2}\Delta_{2}^{3}}\right)}^{2}}{\Delta_{2}\zeta_{2}{\left(\Delta_{2}-\zeta_{2}\right)}^{2}}\\ k_{5}=\frac{2\sqrt{{\left(\sqrt{A_{02}\zeta_{2}^{3}}-\sqrt{A_{2}\Delta_{2}^{3}}\right)}^{2}{\left(\sqrt{A_{02}\zeta_{2}}-\sqrt{A_{2}\Delta_{2}}\right)}^{2}}}{\Delta_{2}\zeta_{2}{\left(\Delta_{2}-\zeta_{2}\right)}^{2}}\\ k_{6}=\frac{A_{02}\zeta_{2}+A_{2}\Delta_{2}-2\sqrt{A_{02}A_{2}\Delta_{2}\zeta_{2}}}{\Delta_{2}\zeta_{2}{\left(\Delta_{2}-\zeta_{2}\right)}^{2}}=\frac{{\left(\sqrt{A_{02}\zeta_{2}}-\sqrt{A_{2}\Delta_{2}}\right)}^{2}}{\Delta_{2}\zeta_{2}{\left(\Delta_{2}-\zeta_{2}\right)}^{2}} \end{array}$$ (30)

or

$$\begin{array}{l} k_{4}=\frac{A_{02}\zeta_{2}^{3}+A_{2}\Delta_{2}^{3}+2\Delta_{2}\zeta_{2}\sqrt{A_{02}A_{2}\Delta_{2}\zeta_{2}}}{\Delta_{2}\zeta_{2}{\left(\Delta_{2}-\zeta_{2}\right)}^{2}}=\frac{{\left(\sqrt{A_{02}\zeta_{2}^{3}}+\sqrt{A_{2}\Delta_{2}^{3}}\right)}^{2}}{\Delta_{2}\zeta_{2}{\left(\Delta_{2}-\zeta_{2}\right)}^{2}}\\ k_{5}=2\frac{A_{02}\zeta_{2}^{2}+A_{2}\Delta_{2}^{2}+(\Delta_{2}+\zeta_{2})\sqrt{A_{02}A_{2}\Delta_{2}\zeta_{2}}}{\Delta_{2}\zeta_{2}{\left(\Delta_{2}-\zeta_{2}\right)}^{2}}\\ k_{6}=\frac{A_{02}\zeta_{2}+A_{2}\Delta_{2}+2\sqrt{A_{02}A_{2}\Delta_{2}\zeta_{2}}}{\Delta_{2}\zeta_{2}{\left(\Delta_{2}-\zeta_{2}\right)}^{2}}=\frac{{\left(\sqrt{A_{02}\zeta_{2}}+\sqrt{A_{2}\Delta_{2}}\right)}^{2}}{\Delta_{2}\zeta_{2}{\left(\Delta_{2}-\zeta_{2}\right)}^{2}} \end{array}$$ (31)

It can be seen from Eqs (30) and (31), *k*_4_ ≥ 0, *k*_5_ ≥ 0, *k*_6_ > 0, and because Δ_2_ ≥ 0, *ζ*_2_ ≥ 0. Therefore, according to Eqs (10) and 11 and $k_{5}=2\sqrt{k_{4}k_{6}}$ and basic inequalities, the range of k5 can be obtained as

$$0\leq k_{5}\leq \frac{A_{02}\zeta_{2}^{2}+A_{2}\Delta_{2}^{2}}{2\Delta_{2}^{2}\zeta_{2}^{2}}$$ (32)

According to Eqs (31) and (32), it can be obtained

$$k_{5}=\frac{2[A_{02}\zeta_{2}^{2}+A_{2}\Delta_{2}^{2}+(\Delta_{2}+\zeta_{2})\sqrt{A_{02}A_{2}\Delta_{2}\zeta_{2}}]}{\Delta_{2}\zeta_{2}{\left(\Delta_{2}-\zeta_{2}\right)}^{2}}\leq \frac{A_{02}\zeta_{2}^{2}+A_{2}\Delta_{2}^{2}}{2\Delta_{2}^{2}\zeta_{2}^{2}}$$

That is

$$\left[4\Delta_{2}\zeta_{2}-{\left(\Delta_{2}-\zeta_{2}\right)}^{2}\right](A_{02}\zeta_{2}^{2}+A_{2}\Delta_{2}^{2})+4\Delta_{2}\zeta_{2}(\Delta_{2}+\zeta_{2})\sqrt{A_{02}A_{2}\Delta_{2}\zeta_{2}}\leq 0$$ (33)

Assume that Δ_2_ ≥ 1, *ζ*_2_ ≥ 1, 0 ≤ Δ_2_ − *ζ*_2_ ≤ 1. In this case, 4Δ_2_*ζ*_2_ ≥ 4, (Δ_2_ − *ζ*_2_)^2^ ≤ 1, so 4Δ_2_*ζ*_2_ − (Δ_2_ − *ζ*_2_)^2^ ≥ 0. At this time

$$\left[4\Delta_{2}\zeta_{2}-{\left(\Delta_{2}-\zeta_{2}\right)}^{2}\right](A_{02}\zeta_{2}^{2}+A_{2}\Delta_{2}^{2})+4\Delta_{2}\zeta_{2}(\Delta_{2}+\zeta_{2})\sqrt{A_{02}A_{2}\Delta_{2}\zeta_{2}}\geq 0$$ (34)

This is contrary to Eq (33), so the solution of the quality loss coefficient corresponding to Eq (31) should be discarded.

*L*_*N*2_(*y*) = 0 has two zero solutions, respectively *y*_6_ = *y*_0_ and *y*_7,8_ = *y*_0_ − *k*_5_/2*k*_6_. Because of *k*_4_ ≥ 0, *k*_5_ ≥ 0, *k*_6_ < 0, so *k*_5_/2*k*_6_ ≥ 0, which is *y*_7,8_ > *y*_1_.

*L*_*N*2_(*y*) calculates the first derivative of y, we can get

$$L_{N2}^{\prime }(y)=k_{4}+k_{5}(y-y_{0})+k_{6}{\left(y-y_{0}\right)}^{2}$$ (35)

**Fig 6:**
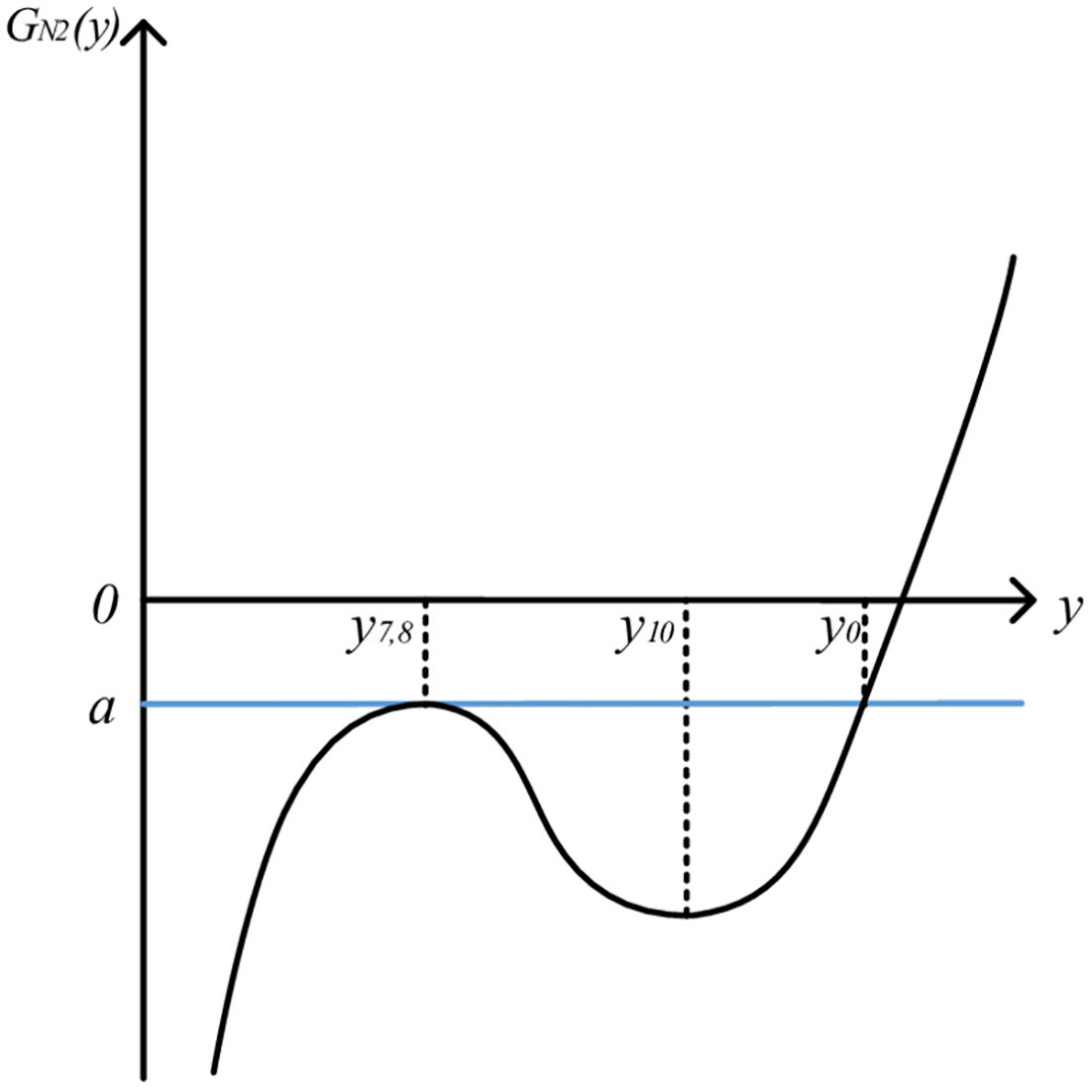
Image of QGLF ($k_{5}=2\sqrt{k_{4}k_{6}}$).

Let $L_{N2}^{\prime }(y)=0$, the solution is *y*_9_ = *y*_0_ − *k*_5_/2*k*_6_ = *y*_2,8_, *y*_10_ = *y*_0_ − *k*_5_/6*k*_6_. Because of *y*_7,8_ < *y*_10_ < *y*_0_, the image of function *L*_*N*2_(*y*) is roughly in the form of " increase, subtraction and increase". In this case, the image of QGLF *G*_*N*2_(*y*) is shown in Fig 6.

From the QGLF image, it can be seen that when [*y*_0_, *y*_0_ + Δ_2_], the function image is monotonous, and the quality gain-loss in this case is consistent with the actual product quality gain-loss. Therefore, when $k_{5}=2\sqrt{k_{4}k_{6}}$, QGLF *G*_*N*2_(*y*) on the interval [*y*_0_, *y*_0_ + Δ_2_] can be formulated as

$$\begin{array}{l} G_{N2}(y)=a+\frac{{\left(\sqrt{A_{02}\zeta_{2}^{3}}-\sqrt{A_{2}\Delta_{2}^{3}}\right)}^{2}}{\Delta_{2}\zeta_{2}{\left(\Delta_{2}-\zeta_{2}\right)}^{2}}(y-y_{0})\\ +\frac{2\sqrt{{\left(\sqrt{A_{02}\zeta_{2}^{3}}-\sqrt{A_{2}\Delta_{2}^{3}}\right)}^{2}{\left(\sqrt{A_{02}\zeta_{2}}-\sqrt{A_{2}\Delta_{2}}\right)}^{2}}}{\Delta_{2}\zeta_{2}{\left(\Delta_{2}-\zeta_{2}\right)}^{2}}{\left(y-y_{0}\right)}^{2}\\ +\frac{{\left(\sqrt{A_{02}\zeta_{2}}-\sqrt{A_{2}\Delta_{2}}\right)}^{2}}{\Delta_{2}\zeta_{2}{\left(\Delta_{2}-\zeta_{2}\right)}^{2}}{\left(y-y_{0}\right)}^{3} \end{array}$$ (36)

**Fig 7:**
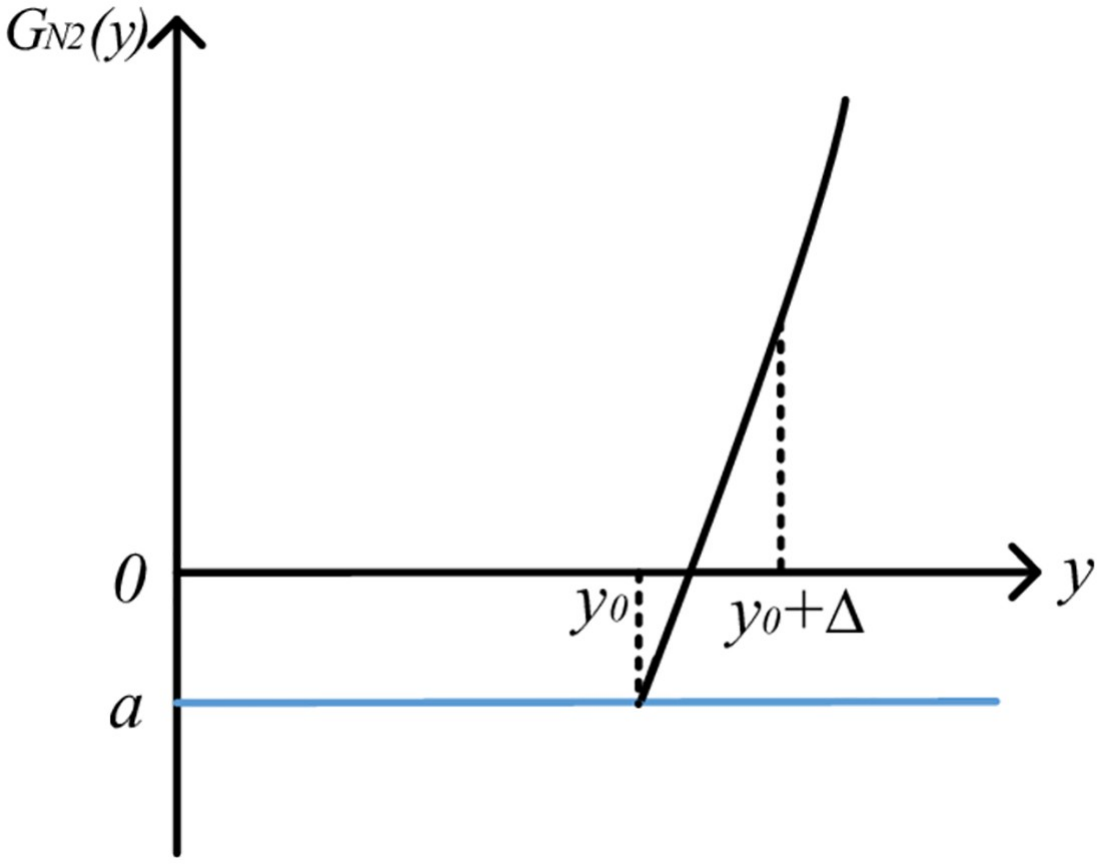
Image of QGLF *G*_*N*2_(*y*) (*y* ∈ [*y*_0_, *y*_0_ + Δ_2_] and $k_{5}=2\sqrt{k_{4}k_{6}}$).

The image is shown in Fig 7.

*3*.*1*.*2*.*2 When $k_{5}=-2\sqrt{k_{4}k_{6}}$*.

According to Eqs (10) and 11 and $k_{5}=-2\sqrt{k_{4}k_{6}}$, it can be solved

$$\begin{array}{l} k_{4}=\frac{{\left(\sqrt{A_{02}\zeta_{2}^{3}}-\sqrt{A_{2}\Delta_{2}^{3}}\right)}^{2}}{\Delta_{2}\zeta_{2}{\left(\Delta_{2}-\zeta_{2}\right)}^{2}}\\ k_{5}=-\frac{2\sqrt{{\left(\sqrt{A_{02}\zeta_{2}^{3}}-\sqrt{A_{2}\Delta_{2}^{3}}\right)}^{2}{\left(\sqrt{A_{02}\zeta_{2}}-\sqrt{A_{2}\Delta_{2}}\right)}^{2}}}{\Delta_{2}\zeta_{2}{\left(\Delta_{2}-\zeta_{2}\right)}^{2}}\\ k_{6}=\frac{{\left(\sqrt{A_{02}\zeta_{2}}-\sqrt{A_{2}\Delta_{2}}\right)}^{2}}{\Delta_{2}\zeta_{2}{\left(\Delta_{2}-\zeta_{2}\right)}^{2}} \end{array}$$ (37)

or

$$\begin{array}{l} k_{4}=\frac{{\left(\sqrt{A_{02}\zeta_{2}^{3}}+\sqrt{A_{2}\Delta_{2}^{3}}\right)}^{2}}{\Delta_{2}\zeta_{2}{\left(\Delta_{2}-\zeta_{2}\right)}^{2}}\\ k_{5}=-2\frac{A_{02}\zeta_{2}^{2}+A_{2}\Delta_{2}^{2}+(\Delta_{2}+\zeta_{2})\sqrt{A_{02}A_{2}\Delta_{2}\zeta_{2}}}{\Delta_{2}\zeta_{2}{\left(\Delta_{2}-\zeta_{2}\right)}^{2}}\\ k_{6}=\frac{{\left(\sqrt{A_{02}\zeta_{2}}+\sqrt{A_{2}\Delta_{2}}\right)}^{2}}{\Delta_{2}\zeta_{2}{\left(\Delta_{2}-\zeta_{2}\right)}^{2}} \end{array}$$ (38)

**Fig 8:**
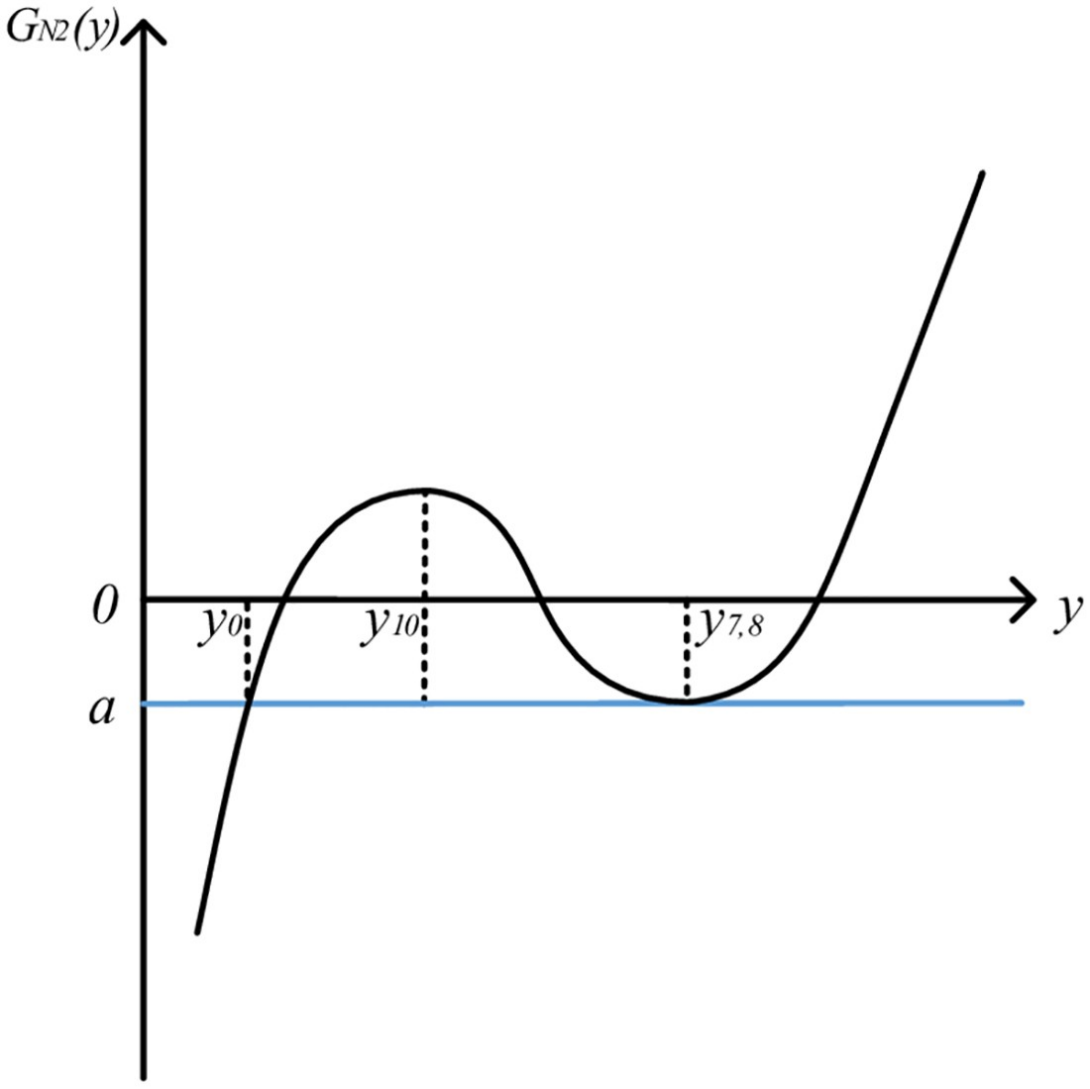
Image of QGLF *G*_N2_(*y*) ($k_{6}=-2\sqrt{k_{4}k_{5}}$).

It can be seen that *k*_4_ ≥ 0, *k*_5_ ≤ 0, *k*_6_ > 0, so *y*_7,8_ > *y*_10_ > *y*_0_. At this time, the image of QGLF is roughly shown in Fig 8.

From the QGLF image, it can be seen that when [*y*_0_, *y*_0_ + Δ_2_], the function image is not monotonous, and the quality gain-loss in this case is not consistent with the actual product quality gain-loss. Therefore, when $k_{5}=-2\sqrt{k_{4}k_{6}}$, the obtained QGLF does not conform to the reality and should be discarded.

To conclude, when the loss of primary term and cubic term is not ignored and the compensation quantity is constant, the asymmetric QGLF can be formulated as

$$G_{N}(y)=\left\{\begin{array}{l} a+A_{01},y<y_{0}-\Delta_{1}\\ a-\frac{{\left(\sqrt{A_{01}\zeta_{1}^{3}}-\sqrt{A_{1}\Delta_{1}^{3}}\right)}^{2}}{\Delta_{1}\zeta_{1}{\left(\Delta_{1}-\zeta_{1}\right)}^{2}}(y-y_{0})+\frac{2\sqrt{{\left(\sqrt{A_{01}\zeta_{1}^{3}}-\sqrt{A_{1}\Delta_{1}^{3}}\right)}^{2}{\left(\sqrt{A_{01}\zeta_{1}}-\sqrt{A_{1}\Delta_{1}}\right)}^{2}}}{\Delta_{1}\zeta_{1}{\left(\Delta_{1}-\zeta_{1}\right)}^{2}}{\left(y-y_{0}\right)}^{2}\\ -\frac{{\left(\sqrt{A_{01}\zeta_{1}}-\sqrt{A_{1}\Delta_{1}}\right)}^{2}}{\Delta_{1}\zeta_{1}{\left(\Delta_{1}-\zeta_{1}\right)}^{2}}{\left(y-y_{0}\right)}^{3},y_{0}-\Delta_{1}\leq y<y_{0}\\ a+\frac{{\left(\sqrt{A_{02}\zeta_{2}^{3}}-\sqrt{A_{2}\Delta_{2}^{3}}\right)}^{2}}{\Delta_{2}\zeta_{2}{\left(\Delta_{2}-\zeta_{2}\right)}^{2}}(y-y_{0})+\frac{2\sqrt{{\left(\sqrt{A_{02}\zeta_{2}^{3}}-\sqrt{A_{2}\Delta_{2}^{3}}\right)}^{2}{\left(\sqrt{A_{02}\zeta_{2}}-\sqrt{A_{2}\Delta_{2}}\right)}^{2}}}{\Delta_{2}\zeta_{2}{\left(\Delta_{2}-\zeta_{2}\right)}^{2}}{\left(y-y_{0}\right)}^{2}\\ +\frac{{\left(\sqrt{A_{02}\zeta_{2}}-\sqrt{A_{2}\Delta_{2}}\right)}^{2}}{\Delta_{2}\zeta_{2}{\left(\Delta_{2}-\zeta_{2}\right)}^{2}}{\left(y-y_{0}\right)}^{3},y_{0}\leq y\leq y_{0}+\Delta_{2}\\ a+A_{02},y>y_{0}+\Delta_{2} \end{array}\right.$$ (39)

**Fig 9:**
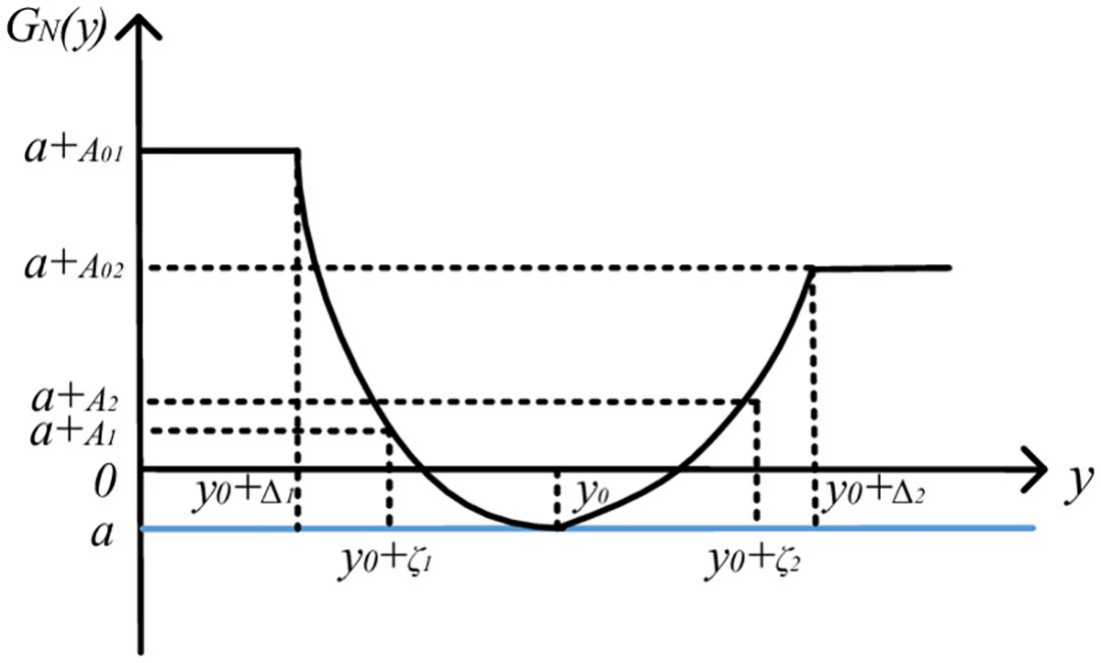
Image of QGLF *G*_*N*_(*y*).

The image of *G*_*N*_(*y*) is shown in Fig 9.

### 3.2 The discriminant is non-negative

#### 3.2.1 When *y* ∈ [*y*_0_ − Δ_1_, *y*_0_)

If $k_{2}^{2}-4k_{1}k_{3}\geq 0$, that is $k_{2}^{2}\geq 4k_{1}k_{3}$. Therefore, the following two cases are discussed, namely *k*_1_*k*_3_ ≤ 0 and $0\leq k_{1}k_{3}\leq \frac{k_{2}^{2}}{4}$.

According to Eqs (8) and (9), it can be solved

$$k_{1}=\frac{A_{01}\zeta_{1}^{3}-A_{1}\Delta_{1}^{3}+k_{2}\Delta_{1}^{2}\zeta_{1}^{2}(\Delta_{1}-\zeta_{1})}{\Delta_{1}\zeta_{1}(\Delta_{1}^{2}-\zeta_{{}_{1}}^{2})}$$

$$k_{3}=\frac{A_{1}\Delta_{1}^{}-A_{01}\zeta_{1}^{}+k_{2}\Delta_{1}^{}\zeta_{1}^{}(\Delta_{1}-\zeta_{1})}{\Delta_{1}\zeta_{1}(\Delta_{1}^{2}-\zeta_{{}_{1}}^{2})}$$

So, *k*_1_*k*_3_ can be expressed as

$$k_{1}k_{3}=\frac{\Delta_{1}\zeta_{1}}{{\left(\Delta_{1}+\zeta_{1}\right)}^{2}}k_{2}^{2}-\frac{A_{01}\zeta_{1}^{2}+A_{1}\Delta_{1}^{2}}{\Delta_{1}\zeta_{1}{\left(\Delta_{1}+\zeta_{1}\right)}^{2}}k_{2}+\frac{A_{01}A_{1}\Delta_{1}\zeta_{1}(\Delta_{1}^{2}+\zeta_{1}^{2})-A_{01}^{2}\zeta_{1}^{4}-A_{1}^{2}\Delta_{1}^{4}}{\Delta_{1}^{2}\zeta_{1}^{2}{\left(\Delta_{1}^{2}-\zeta_{1}^{2}\right)}^{2}}$$ (40)

It can be seen from Eq (40) that *k*_*1*_*k*_*3*_ can be regarded as a function of *k*_*2*_.

*3*.*2*.*1*.*1 When k*_1_*k*_3_ ≤ 0.

According to Eq (40), the value range of *k*_*2*_ can be solved as

$$\frac{(\Delta_{1}-\zeta_{1})(A_{01}\zeta_{1}^{2}+A_{1}\Delta_{1}^{2})-(\Delta_{1}+\zeta_{1})\sqrt{{\left(A_{01}\zeta_{1}^{2}-A_{1}\Delta_{1}^{2}\right)}^{2}}}{2\Delta_{1}^{2}\zeta_{1}^{2}(\Delta_{1}-\zeta_{1})}\leq k_{2}\leq \frac{(\Delta_{1}-\zeta_{1})(A_{01}\zeta_{1}^{2}+A_{1}\Delta_{1}^{2})+(\Delta_{1}+\zeta_{1})\sqrt{{\left(A_{01}\zeta_{1}^{2}-A_{1}\Delta_{1}^{2}\right)}^{2}}}{2\Delta_{1}^{2}\zeta_{1}^{2}(\Delta_{1}-\zeta_{1})}$$ (41)

Since the values of $A_{01}\zeta_{1}^{2}$ and $A_{1}\Delta_{1}^{2}$ cannot be determined, the results in three cases can be obtained by judging the values of the two.

If $A_{01}\zeta_{1}^{2}=A_{1}\Delta_{1}^{2}$, Eq (41) becomes $\frac{A_{01}\zeta_{1}^{2}+A_{1}\Delta_{1}^{2}}{2\Delta_{1}^{2}\zeta_{1}^{2}}\leq k_{2}\leq \frac{A_{01}\zeta_{1}^{2}+A_{1}\Delta_{1}^{2}}{2\Delta_{1}^{2}\zeta_{1}^{2}}$,

That is $k_{2}=\frac{A_{01}\zeta_{1}^{2}+A_{1}\Delta_{1}^{2}}{2\Delta_{1}^{2}\zeta_{1}^{2}}=\frac{2A_{1}\Delta_{1}^{2}}{2\Delta_{1}^{2}\zeta_{1}^{2}}=\frac{A_{1}}{\zeta_{1}^{2}}$ or $k_{2}=\frac{2A_{01}\zeta_{1}^{2}}{2\Delta_{1}^{2}\zeta_{1}^{2}}=\frac{A_{01}}{\Delta_{1}^{2}}$. At this time, the loss of primary and cubic term is 0, and the original formula becomes the quadratic QGLF;

If $A_{01}\zeta_{1}^{2}>A_{1}\Delta_{1}^{2}$, Eq (41) becomes $\frac{A_{1}\Delta_{1}^{3}-A_{01}\zeta_{1}^{3}}{\Delta_{1}^{2}\zeta_{1}^{2}(\Delta_{1}-\zeta_{1})}\leq k_{2}\leq \frac{A_{01}\zeta_{1}^{}-A_{1}\Delta_{1}^{}}{\Delta_{1}^{}\zeta_{1}^{}(\Delta_{1}-\zeta_{1})}$;

If $A_{01}\zeta_{1}^{2}<A_{1}\Delta_{1}^{2}$, Eq (41) becomes $\frac{A_{01}\zeta_{1}^{}-A_{1}\Delta_{1}^{}}{\Delta_{1}^{}\zeta_{1}^{}(\Delta_{1}-\zeta_{1})}\leq k_{2}\leq \frac{A_{1}\Delta_{1}^{3}-A_{01}\zeta_{1}^{3}}{\Delta_{1}^{2}\zeta_{1}^{2}(\Delta_{1}-\zeta_{1})}$.

At this time, QGLF *G*_*N*1_(*y*) can be expressed as

$$\begin{array}{ll} G_{N1}(y) & =a+\frac{A_{01}\zeta_{1}^{3}-A_{1}\Delta_{1}^{3}+k_{2}\Delta_{1}^{2}\zeta_{1}^{2}(\Delta_{1}-\zeta_{1})}{\Delta_{1}\zeta_{1}(\Delta_{1}^{2}-\zeta_{{}_{1}}^{2})}(y-y_{0})+k_{2}{\left(y-y_{0}\right)}^{2}\\ & +\frac{A_{1}\Delta_{1}^{}-A_{01}\zeta_{1}^{}+k_{2}\Delta_{1}^{}\zeta_{1}^{}(\Delta_{1}-\zeta_{1})}{\Delta_{1}\zeta_{1}(\Delta_{1}^{2}-\zeta_{{}_{1}}^{2})}{\left(y-y_{0}\right)}^{3} \end{array}$$ (42)

*3*.*2*.*1*.*2 When k*_1_*k*_3_ ≥ 0.

According to Eq (40), the value range of *k*_*2*_ can be solved as

$$\begin{array}{c} k_{2}\leq \frac{(\Delta_{1}-\zeta_{1})(A_{01}\zeta_{1}^{2}+A_{1}\Delta_{1}^{2})-(\Delta_{1}+\zeta_{1})\sqrt{{\left(A_{01}\zeta_{1}^{2}-A_{1}\Delta_{1}^{2}\right)}^{2}}}{2\Delta_{1}^{2}\zeta_{1}^{2}(\Delta_{1}-\zeta_{1})}\\ \text{Or}\\ k_{2}\geq \frac{(\Delta_{1}-\zeta_{1})(A_{01}\zeta_{1}^{2}+A_{1}\Delta_{1}^{2})+(\Delta_{1}+\zeta_{1})\sqrt{{\left(A_{01}\zeta_{1}^{2}-A_{1}\Delta_{1}^{2}\right)}^{2}}}{2\Delta_{1}^{2}\zeta_{1}^{2}(\Delta_{1}-\zeta_{1})} \end{array}$$ (43)

Same as above analysis, if $A_{01}\zeta_{1}^{2}>A_{1}\Delta_{1}^{2}$, Eq (43) becomes

$$k_{2}\leq \frac{A_{1}\Delta_{1}^{3}-A_{01}\zeta_{1}^{3}}{\Delta_{1}^{2}\zeta_{1}^{2}(\Delta_{1}-\zeta_{1})}\;\text{or}\;k_{2}\geq \frac{A_{01}\zeta_{1}^{}-A_{1}\Delta_{1}^{}}{\Delta_{1}^{}\zeta_{1}^{}(\Delta_{1}-\zeta_{1})}$$ (44)

If $A_{01}\zeta_{1}^{2}<A_{1}\Delta_{1}^{2}$, Eq (43) becomes

$$k_{2}\leq \frac{A_{01}\zeta_{1}^{}-A_{1}\Delta_{1}^{}}{\Delta_{1}^{}\zeta_{1}^{}(\Delta_{1}-\zeta_{1})}\;\text{or}\;k_{2}\geq \frac{A_{1}\Delta_{1}^{3}-A_{01}\zeta_{1}^{3}}{\Delta_{1}^{2}\zeta_{1}^{2}(\Delta_{1}-\zeta_{1})}$$ (45)

*3*.*2*.*1*.*3 When $k_{1}k_{3}\leq \frac{k_{2}^{2}}{4}$*.

That is

$$-\frac{{\left(\Delta_{1}-\zeta_{1}\right)}^{2}}{4{\left(\Delta_{1}+\zeta_{1}\right)}^{2}}k_{2}^{2}-\frac{A_{01}\zeta_{1}^{2}+A_{1}\Delta_{1}^{2}}{\Delta_{1}\zeta_{1}{\left(\Delta_{1}+\zeta_{1}\right)}^{2}}k_{2}+\frac{A_{01}A_{1}\Delta_{1}\zeta_{1}(\Delta_{1}^{2}+\zeta_{1}^{2})-A_{01}^{2}\zeta_{1}^{4}-A_{1}^{2}\Delta_{1}^{4}}{\Delta_{1}^{2}\zeta_{1}^{2}{\left(\Delta_{1}^{2}-\zeta_{1}^{2}\right)}^{2}}\leq 0$$ (46)

According to Eq (41), the value range of *k*_*2*_ can be solved as

$$k_{2}\leq -\frac{2[A_{01}\zeta_{1}^{2}+A_{1}\Delta_{1}^{2}+(\Delta_{1}+\zeta_{1})\sqrt{A_{01}A_{1}\Delta_{1}\zeta_{1}}]}{\Delta_{1}\zeta_{1}{\left(\Delta_{1}-\zeta_{1}\right)}^{2}}\;\text{or}\;k_{2}\geq -\frac{2[A_{01}\zeta_{1}^{2}+A_{1}\Delta_{1}^{2}-(\Delta_{1}+\zeta_{1})\sqrt{A_{01}A_{1}\Delta_{1}\zeta_{1}}]}{\Delta_{1}\zeta_{1}{\left(\Delta_{1}-\zeta_{1}\right)}^{2}}$$ (47)

Therefore, according to Eqs (44) and (47), when $A_{01}\zeta_{1}^{2}>A_{1}\Delta_{1}^{2}$, the value range of *k*_*2*_ is

$$\begin{array}{l} \left(-\infty ,-\frac{2[A_{01}\zeta_{1}^{2}+A_{1}\Delta_{1}^{2}+(\Delta_{1}+\zeta_{1})\sqrt{A_{01}A_{1}\Delta_{1}\zeta_{1}}]}{\Delta_{1}\zeta_{1}{\left(\Delta_{1}-\zeta_{1}\right)}^{2}}\right]\\ \cup [-\frac{2[A_{01}\zeta_{1}^{2}+A_{1}\Delta_{1}^{2}-(\Delta_{1}+\zeta_{1})\sqrt{A_{01}A_{1}\Delta_{1}\zeta_{1}}]}{\Delta_{1}\zeta_{1}{\left(\Delta_{1}-\zeta_{1}\right)}^{2}},\frac{A_{1}\Delta_{1}^{3}-A_{01}\zeta_{1}^{3}}{\Delta_{1}^{2}\zeta_{1}^{2}(\Delta_{1}-\zeta_{1})}]\\ \cup [\frac{A_{01}\zeta_{1}^{}-A_{1}\Delta_{1}^{}}{\Delta_{1}^{}\zeta_{1}^{}(\Delta_{1}-\zeta_{1})},+\infty ) \end{array}$$ (48)

According to Eqs (45) and (47), when $A_{01}\zeta_{1}^{2}<A_{1}\Delta_{1}^{2}$, the value range of *k*_*2*_ is

$$\begin{array}{l} \left(-\infty ,-\frac{2[A_{01}\zeta_{1}^{2}+A_{1}\Delta_{1}^{2}+(\Delta_{1}+\zeta_{1})\sqrt{A_{01}A_{1}\Delta_{1}\zeta_{1}}]}{\Delta_{1}\zeta_{1}{\left(\Delta_{1}-\zeta_{1}\right)}^{2}}\right]\\ \cup [-\frac{2[A_{01}\zeta_{1}^{2}+A_{1}\Delta_{1}^{2}-(\Delta_{1}+\zeta_{1})\sqrt{A_{01}A_{1}\Delta_{1}\zeta_{1}}]}{\Delta_{1}\zeta_{1}{\left(\Delta_{1}-\zeta_{1}\right)}^{2}},\frac{A_{01}\zeta_{1}^{}-A_{1}\Delta_{1}^{}}{\Delta_{1}^{}\zeta_{1}^{}(\Delta_{1}-\zeta_{1})}]\\ \cup [\frac{A_{1}\Delta_{1}^{3}-A_{01}\zeta_{1}^{3}}{\Delta_{1}^{2}\zeta_{1}^{2}(\Delta_{1}-\zeta_{1})},+\infty ) \end{array}$$ (49)

In summary, when *y* ∈ [*y*_0_ − Δ_1_, *y*_0_), the QGLF without ignoring the loss of primary and cubic term and with constant compensation quantity can be expressed as

$$\begin{array}{ll} G_{N1}(y) & =a+\frac{A_{01}\zeta_{1}^{3}-A_{1}\Delta_{1}^{3}+k_{2}\Delta_{1}^{2}\zeta_{1}^{2}(\Delta_{1}-\zeta_{1})}{\Delta_{1}\zeta_{1}(\Delta_{1}^{2}-\zeta_{{}_{1}}^{2})}(y-y_{0})\\ & +k_{2}{\left(y-y_{0}\right)}^{2}+\frac{A_{1}\Delta_{1}^{}-A_{01}\zeta_{1}^{}+k_{2}\Delta_{1}^{}\zeta_{1}^{}(\Delta_{1}-\zeta_{1})}{\Delta_{1}\zeta_{1}(\Delta_{1}^{2}-\zeta_{{}_{1}}^{2})}{\left(y-y_{0}\right)}^{3} \end{array}$$ (50)

The value range of *k*_*2*_ is analyzed above.

#### 3.2.2 When *y* ∈ [*y*_0_, *y*_0_ + Δ_2_}

Same as 3.2.1 analysis procedure. If $k_{5}^{2}-4k_{4}k_{6}\geq 0$, that is $k_{5}^{2}\geq 4k_{4}k_{6}$, the discussion is divided into two cases, namely *k*_4_*k*_6_ ≤ 0 and $0\leq k_{4}k_{6}\leq \frac{k_{5}^{2}}{4}$.

According to Eqs (10) and (11), it can be solved

$$k_{4}=\frac{A_{2}\Delta_{2}^{3}-A_{02}\zeta_{2}^{3}-k_{5}\Delta_{2}^{2}\zeta_{2}^{2}(\Delta_{2}-\zeta_{2})}{\Delta_{2}\zeta_{2}(\Delta_{2}^{2}-\zeta_{2}^{2})}$$

$$k_{6}=\frac{A_{02}\zeta_{2}^{}-A_{2}\Delta_{2}^{}-k_{5}\Delta_{2}^{}\zeta_{2}^{}(\Delta_{2}-\zeta_{2})}{\Delta_{2}\zeta_{2}(\Delta_{2}^{2}-\zeta_{2}^{2})}$$

So, *k*_4_*k*_6_ can be expressed as

$$k_{4}k_{6}=\frac{\Delta_{2}\zeta_{2}}{{\left(\Delta_{2}+\zeta_{2}\right)}^{2}}k_{5}^{2}-\frac{A_{2}\Delta_{2}^{2}+A_{02}\zeta_{2}^{2}}{\Delta_{2}\zeta_{2}{\left(\Delta_{2}+\zeta_{2}\right)}^{2}}k_{5}+\frac{A_{02}A_{2}\Delta_{2}\zeta_{2}(\Delta_{2}^{2}+\zeta_{2}^{2})-A_{02}^{2}\zeta_{2}^{4}-A_{2}^{2}\Delta_{2}^{4}}{\Delta_{2}^{2}\zeta_{2}^{2}{\left(\Delta_{2}^{2}-\zeta_{2}^{2}\right)}^{2}}$$ (51)

It can be seen from Eq (51) that *k*_*4*_*k*_*6*_ can be regarded as a function of *k*_*5*_.

*3*.*2*.*2*.*1 When k*_4_*k*_6_ ≤ 0.

The value range of *k*_*5*_ can be solved according to Eq (51)

$$\frac{(\Delta_{2}-\zeta_{2})(A_{02}\zeta_{2}^{2}+A_{2}\Delta_{2}^{2})-(\Delta_{2}+\zeta_{2})\sqrt{{\left(A_{02}\zeta_{2}^{2}-A_{2}\Delta_{2}^{2}\right)}^{2}}}{2\Delta_{2}^{2}\zeta_{2}^{2}(\Delta_{2}-\zeta_{2})}\leq k_{5}\leq \frac{(\Delta_{2}-\zeta_{2})(A_{02}\zeta_{2}^{2}+A_{2}\Delta_{2}^{2})+(\Delta_{2}+\zeta_{2})\sqrt{{\left(A_{02}\zeta_{2}^{2}-A_{2}\Delta_{2}^{2}\right)}^{2}}}{2\Delta_{2}^{2}\zeta_{2}^{2}(\Delta_{2}-\zeta_{2})}$$ (52)

Since the values of $A_{02}\zeta_{2}^{2}$ and $A_{2}\Delta_{2}^{2}$ cannot be determined, the results in three cases can be obtained by judging the values of the two.

If $A_{02}\zeta_{2}^{2}=A_{2}\Delta_{2}^{2}$, Eq (52) becomes $\frac{A_{02}\zeta_{2}^{2}+A_{2}\Delta_{2}^{2}}{2\Delta_{2}^{2}\zeta_{2}^{2}}\leq k_{5}\leq \frac{A_{02}\zeta_{2}^{2}+A_{2}\Delta_{2}^{2}}{2\Delta_{2}^{2}\zeta_{2}^{2}}$, That is $k_{5}=\frac{2A_{2}\Delta_{2}^{2}}{2\Delta_{2}^{2}\zeta_{2}^{2}}=\frac{A_{2}}{\zeta_{2}^{2}}$ or $k_{5}=\frac{2A_{02}\zeta_{2}^{2}}{2\Delta_{2}^{2}\zeta_{2}^{2}}=\frac{A_{02}}{\Delta_{2}^{2}}$. At this time, the loss of primary and cubic term is 0, and the original formula becomes the quadratic QGLF.

If $A_{02}\zeta_{2}^{2}>A_{2}\Delta_{2}^{2}$, Eq (52) becomes $\frac{A_{2}\Delta_{2}^{3}-A_{02}\zeta_{2}^{3}}{\Delta_{2}^{2}\zeta_{2}^{2}(\Delta_{2}-\zeta_{2})}\leq k_{5}\leq \frac{A_{02}\zeta_{2}^{}-A_{2}\Delta_{2}^{}}{\Delta_{2}^{}\zeta_{2}^{}(\Delta_{2}-\zeta_{2})}$;

If $A_{02}\zeta_{2}^{2}<A_{2}\Delta_{2}^{2}$, Eq (52) becomes $\frac{A_{02}\zeta_{2}^{}-A_{2}\Delta_{2}^{}}{\Delta_{2}^{}\zeta_{2}^{}(\Delta_{2}-\zeta_{2})}\leq k_{5}\leq \frac{A_{2}\Delta_{2}^{3}-A_{02}\zeta_{2}^{3}}{\Delta_{2}^{2}\zeta_{2}^{2}(\Delta_{2}-\zeta_{2})}$.

At this time, QGLF *G*_*N*2_(*y*) can be expressed as

$$\begin{array}{ll} G_{N2}(y) & =a+\frac{A_{2}\Delta_{2}^{3}-A_{02}\zeta_{2}^{3}-k_{5}\Delta_{2}^{2}\zeta_{2}^{2}(\Delta_{2}-\zeta_{2})}{\Delta_{2}\zeta_{2}(\Delta_{2}^{2}-\zeta_{2}^{2})}(y-y_{0})+k_{5}{\left(y-y_{0}\right)}^{2}\\ & +\frac{A_{02}\zeta_{2}^{}-A_{2}\Delta_{2}^{}-k_{5}\Delta_{2}^{}\zeta_{2}^{}(\Delta_{2}-\zeta_{2})}{\Delta_{2}\zeta_{2}(\Delta_{2}^{2}-\zeta_{2}^{2})}{\left(y-y_{0}\right)}^{3} \end{array}$$ (53)

*3*.*2*.*2*.*2 When k*_4_*k*_6_ ≥ 0.

The value range of *k*_*5*_ can be solved according to Eq (51)

$$\begin{array}{c} k_{5}\leq \frac{(\Delta_{2}-\zeta_{2})(A_{02}\zeta_{2}^{2}+A_{2}\Delta_{2}^{2})-(\Delta_{2}+\zeta_{2})\sqrt{{\left(A_{02}\zeta_{2}^{2}-A_{2}\Delta_{2}^{2}\right)}^{2}}}{2\Delta_{2}^{2}\zeta_{2}^{2}(\Delta_{2}-\zeta_{2})}\\ \text{or}\\ k_{5}\geq \frac{(\Delta_{2}-\zeta_{2})(A_{02}\zeta_{2}^{2}+A_{2}\Delta_{2}^{2})+(\Delta_{2}+\zeta_{2})\sqrt{{\left(A_{02}\zeta_{2}^{2}-A_{2}\Delta_{2}^{2}\right)}^{2}}}{2\Delta_{2}^{2}\zeta_{2}^{2}(\Delta_{2}-\zeta_{2})} \end{array}$$ (54)

Same as above analysis, if $A_{02}\zeta_{2}^{2}>A_{2}\Delta_{2}^{2}$, Eq (54) becomes

$$k_{5}\leq \frac{A_{2}\Delta_{2}^{3}-A_{02}\zeta_{2}^{3}}{\Delta_{2}^{2}\zeta_{2}^{2}(\Delta_{2}-\zeta_{2})}\;\text{or}\;k_{5}\geq \frac{A_{02}\zeta_{2}^{}-A_{2}\Delta_{2}^{}}{\Delta_{2}^{}\zeta_{2}^{}(\Delta_{2}-\zeta_{2})}$$ (55)

If $A_{02}\zeta_{2}^{2}<A_{2}\Delta_{2}^{2}$, Eq (54) becomes

$$k_{5}\leq \frac{A_{02}\zeta_{2}^{}-A_{2}\Delta_{2}^{}}{\Delta_{2}^{}\zeta_{2}^{}(\Delta_{2}-\zeta_{2})}\;\text{or}\;k_{5}\geq \frac{A_{2}\Delta_{2}^{3}-A_{02}\zeta_{2}^{3}}{\Delta_{2}^{2}\zeta_{2}^{2}(\Delta_{2}-\zeta_{2})}$$ (56)

*3*.*2*.*2*.*3 When $k_{4}k_{6}\leq \frac{k_{5}^{2}}{4}$*.

That is

$$-\frac{{\left(\Delta_{2}-\zeta_{2}\right)}^{2}}{4{\left(\Delta_{2}+\zeta_{2}\right)}^{2}}k_{5}^{2}-\frac{A_{2}\Delta_{2}^{2}+A_{02}\zeta_{2}^{2}}{\Delta_{2}\zeta_{2}{\left(\Delta_{2}+\zeta_{2}\right)}^{2}}k_{5}+\frac{A_{02}A_{2}\Delta_{2}\zeta_{2}(\Delta_{2}^{2}+\zeta_{2}^{2})-A_{02}^{2}\zeta_{2}^{4}-A_{2}^{2}\Delta_{2}^{4}}{\Delta_{2}^{2}\zeta_{2}^{2}{\left(\Delta_{2}^{2}-\zeta_{2}^{2}\right)}^{2}}\leq 0$$ (57)

The value range of *k*_*5*_ can be obtained from Eq (57)

$$k_{5}\leq -\frac{2[A_{02}\zeta_{2}^{2}+A_{2}\Delta_{2}^{2}+(\Delta_{2}+\zeta_{2})\sqrt{A_{02}A_{2}\Delta_{2}\zeta_{2}}]}{\Delta_{2}\zeta_{2}{\left(\Delta_{2}-\zeta_{2}\right)}^{2}}\;\text{or}\;k_{5}\geq -\frac{2[A_{02}\zeta_{2}^{2}+A_{2}\Delta_{2}^{2}-(\Delta_{2}+\zeta_{2})\sqrt{A_{02}A_{2}\Delta_{2}\zeta_{2}}]}{\Delta_{2}\zeta_{2}{\left(\Delta_{2}-\zeta_{2}\right)}^{2}}$$ (58)

Therefore, according to Eqs (55) and (58), when $A_{02}\zeta_{2}^{2}>A_{2}\Delta_{2}^{2}$, the value range of *k*_*5*_ is

$$\begin{array}{l} \left(-\infty ,-\frac{2[A_{02}\zeta_{2}^{2}+A_{2}\Delta_{2}^{2}+(\Delta_{2}+\zeta_{2})\sqrt{A_{02}A_{2}\Delta_{2}\zeta_{2}}]}{\Delta_{2}\zeta_{2}{\left(\Delta_{2}-\zeta_{2}\right)}^{2}}\right]\\ \cup [-\frac{2[A_{02}\zeta_{2}^{2}+A_{2}\Delta_{2}^{2}-(\Delta_{2}+\zeta_{2})\sqrt{A_{02}A_{2}\Delta_{2}\zeta_{2}}]}{\Delta_{2}\zeta_{2}{\left(\Delta_{2}-\zeta_{2}\right)}^{2}},\frac{A_{2}\Delta_{2}^{3}-A_{02}\zeta_{2}^{3}}{\Delta_{2}^{2}\zeta_{2}^{2}(\Delta_{2}-\zeta_{2})}]\\ \cup [\frac{A_{02}\zeta_{2}^{}-A_{2}\Delta_{2}^{}}{\Delta_{2}^{}\zeta_{2}^{}(\Delta_{2}-\zeta_{2})},+\infty ) \end{array}$$ (59)

According to Eqs (56) and (58), when $A_{02}\zeta_{2}^{2}<A_{2}\Delta_{2}^{2}$, the value range of *k*_*5*_ is

$$\begin{array}{l} \left(-\infty ,-\frac{2[A_{02}\zeta_{2}^{2}+A_{2}\Delta_{2}^{2}+(\Delta_{2}+\zeta_{2})\sqrt{A_{02}A_{2}\Delta_{2}\zeta_{2}}]}{\Delta_{2}\zeta_{2}{\left(\Delta_{2}-\zeta_{2}\right)}^{2}}\right]\\ \cup [-\frac{2[A_{02}\zeta_{2}^{2}+A_{2}\Delta_{2}^{2}-(\Delta_{2}+\zeta_{2})\sqrt{A_{02}A_{2}\Delta_{2}\zeta_{2}}]}{\Delta_{2}\zeta_{2}{\left(\Delta_{2}-\zeta_{2}\right)}^{2}},\frac{A_{02}\zeta_{2}^{}-A_{2}\Delta_{2}^{}}{\Delta_{2}^{}\zeta_{2}^{}(\Delta_{2}-\zeta_{2})}]\\ \cup [\frac{A_{2}\Delta_{2}^{3}-A_{02}\zeta_{2}^{3}}{\Delta_{2}^{2}\zeta_{2}^{2}(\Delta_{2}-\zeta_{2})},+\infty ) \end{array}$$ (60)

In summary, when *y* ∈ [*y*_0_, *y*_0_ + Δ_2_], the QGLF without ignoring the loss of primary and cubic term and with constant compensation quantity can be expressed as

$$\begin{array}{ll} G_{N2}(y) & =a+\frac{A_{2}\Delta_{2}^{3}-A_{02}\zeta_{2}^{3}-k_{5}\Delta_{2}^{2}\zeta_{2}^{2}(\Delta_{2}-\zeta_{2})}{\Delta_{2}\zeta_{2}(\Delta_{2}^{2}-\zeta_{2}^{2})}(y-y_{0})+k_{5}{\left(y-y_{0}\right)}^{2}\\ & +\frac{A_{02}\zeta_{2}^{}-A_{2}\Delta_{2}^{}-k_{5}\Delta_{2}^{}\zeta_{2}^{}(\Delta_{2}-\zeta_{2})}{\Delta_{2}\zeta_{2}(\Delta_{2}^{2}-\zeta_{2}^{2})}{\left(y-y_{0}\right)}^{3} \end{array}$$ (61)

The value range of *k*_*5*_ is described above.

According to the above analysis, the QGLF without ignoring the loss of primary and cubic term and with constant compensation quantity can be expressed as

$$G_{N}(y)=\left\{\begin{array}{ll} a+A_{01} & ,y<y_{0}-\Delta_{1}\\ a+\frac{A_{01}\zeta_{1}^{3}-A_{1}\Delta_{1}^{3}+k_{2}\Delta_{1}^{2}\zeta_{1}^{2}(\Delta_{1}-\zeta_{1})}{\Delta_{1}\zeta_{1}(\Delta_{1}^{2}-\zeta_{{}_{1}}^{2})}(y-y_{0}) & \\ \;+k_{2}{\left(y-y_{0}\right)}^{2}+\frac{A_{1}\Delta_{1}^{}-A_{01}\zeta_{1}^{}+k_{2}\Delta_{1}^{}\zeta_{1}^{}(\Delta_{1}-\zeta_{1})}{\Delta_{1}\zeta_{1}(\Delta_{1}^{2}-\zeta_{{}_{1}}^{2})}{\left(y-y_{0}\right)}^{3} & ,y_{0}-\Delta_{1}\leq y<y_{0}\\ a+\frac{A_{\text{2}}\Delta_{\text{2}}^{3}-A_{\text{02}}\zeta_{\text{2}}^{3}-k_{5}\Delta_{2}^{2}\zeta_{2}^{2}(\Delta_{2}-\zeta_{2})}{\Delta_{2}\zeta_{2}(\Delta_{2}^{2}-\zeta_{2}^{2})}(y-y_{0}) & \\ \;+k_{5}{\left(y-y_{0}\right)}^{2}+\frac{A_{02}\zeta_{2}^{}-A_{2}\Delta_{2}^{}-k_{5}\Delta_{2}^{}\zeta_{2}^{}(\Delta_{2}-\zeta_{2})}{\Delta_{2}\zeta_{2}(\Delta_{2}^{2}-\zeta_{2}^{2})}{\left(y-y_{0}\right)}^{3} & ,y_{0}\leq y\leq y_{0}+\Delta_{2}\\ a+A_{02} & ,y>y_{0}+\Delta_{2} \end{array}\right.$$ (62)

## 4 Case analysis

Dam concrete construction mainly includes concrete production, concrete transportation, concrete pouring and concrete maintenance and so on. Among them, the key quality index of concrete production has concrete mixture out of the machine slump (unit: cm) for Nominal-the-type characteristic. Slump refers to the slump height of concrete under the action of its own gravity under certain conditions, and is an important parameter used to describe the fluidity and plasticity of concrete. After mixing concrete, it usually needs to be poured by conveyor belt or pumping, which will cause slump loss in the process. If the slump at the outlet is too small, it will cause difficulty in concrete placement and affect the concrete pouring speed. If the workability fails to meet the requirements, it must be treated as waste; if the slump at the outlet is too large, the dispersion of concrete aggregate will be large, which will affect the concrete strength. Therefore, it is very important to control the concrete slump reasonably.

This example is based on the "concrete quality control standards" (GB50164-2011) in the concrete mixture out of the machine slump standards, combined with a dam concrete production project design specifications and design requirements. The calculation process of quality gain-loss that does not neglect the loss of primary and cubic term loss is explained. The quality characteristic target value is 12cm, the quality loss is 48 RMB /m³ when the outlet slump is 10cm, and the quality loss is 35 RMB /m³ when the outlet slump is 14cm. When the outlet slump is 7cm, the concrete pouring workability does not meet the requirements, and the quality loss is 300 RMB/m³. When the outlet slump is 17cm, the aggregate is easy to disperse in the conveying process, resulting in the reduction of concrete strength, and the quality loss is 220 RMB/m³. Assume that the amount of compensation generated by the next process for the quality compensation of this process or the cooperation between parallel processes is 20 RMB/m³. In order to evaluate the production quality of dam concrete, 10 concrete mixture samples were randomly selected and their slumps were tested as 10.8, 12.6, 11.5, 13.3, 13.7, 10.2, 11.0, 10.1, 13.7, 13.9 (unit: cm). The quality of concrete production is evaluated by the quadratic QGLF and the QGLF without ignoring the loss of primary and cubic term, respectively.

(1) Quality gain-loss ignoring the loss of primary and cubic term

According to *y*_0_ = 12, Δ_1_ = Δ_2_ = 5, *A*_01_ = 300, *A*_02_ = 220, *a* = −20, the quadratic QGLF is

$$G_{N}(y)=\left\{\begin{array}{ll} 280 & ,y<7\\ -20+12{\left(y-12\right)}^{2} & ,7\leq y\leq 12\\ -20+8.75{\left(y-12\right)}^{2} & ,12\leq y\leq 17\\ 200 & ,y>17 \end{array}\right.$$ (63)

By substituting the sampled data into the above equation and calculating the arithmetic average, the average quality profit and loss can be obtained: *G*_*Na*_ = 1.458 RMB/m^3^, in which the secondary average quality loss: *G*_*La*_ = 21.458 RMB/m^3^.

(2) Quality gain-loss without ignoring the loss of primary and cubic term

According to *y*_0_ = 12, *ζ*_1_ = *ζ*_*2*_ = 2, Δ_1_ = Δ_2_ = 5, *A*_01_ = 300, *A*_02_ = 220, *A*_1_ = 48, *A*_2_ = 35 *a* = −20 the cubic QGLF is

$$G_{N}(y)=\left\{\begin{array}{ll} 280 & ,y<7\\ -20-9.00593(y-12)+5.69585{\left(y-12\right)}^{2}-0.9006{\left(y-12\right)}^{3} & ,7\leq y<12\\ -20+6.5025026(y-12)+4.16491{\left(y-12\right)}^{2}+0.66692{\left(y-12\right)}^{3} & ,12\leq y\leq 17\\ 200 & ,y>17 \end{array}\right.$$ (64)

The extracted sample data are substituted into the above equation respectively, and then the arithmetic average can be obtained to obtain the average quality gain-loss: *G*_*Nb*_ = 3.317659 RMB/m^3^, among which the primary term loss: *L*_*N*1_ = 10.4456 RMB/m^3^, the quadratic term loss: *L*_*N*2_ = 10.1985 RMB/m^3^ and the cubic term loss: *L*_*N*3_ = 2.67356 RMB/m^3^.

When the quality compensation quantity is constant, the quality gain-loss value of the primary term and the cubic term is 1.85966 RMB/m³ more than that of QGLF of the quadratic term, which is because the quadratic term QGLF ignores the cubic term loss.

As can be seen from the above example, the quadratic term loss is not as big as the primary term loss, so the primary term loss cannot be ignored. The cubic term loss accounts for 12% of the total quality loss, so the cubic term loss cannot be ignored.

## 5 Conclusion

QGLF is represented only by the compensation function and the loss of the quadratic term, ignoring not only the loss of the primary term, but also the higher order term above the quadratic term. This makes the calculated value of QGLF deviate from the actual value, and this deviation can only be reduced but not eliminated which, in part, is inconsistent with the actual situation. This paper studies the cubic QGLF of the Nominal-the-type characteristic from two perspectives of theory and practical application, and puts forward the form of the cubic QGLF of the Nominal-the-type characteristic when the loss of primary and cubic term is not ignored and the compensation quantity is constant, and the corresponding calculation formula of quality loss coefficient is put forward. The cubic QGLF of the Nominal-the-type characteristic model more comprehensively considers the losses of different orders, widens the application range of QGLF, improves the calculation accuracy of quality gain-loss, helps to more accurately assess the relationship between the product quality and the target value, and verifies the feasibility of the model through an example.
